# Cis-TERN: a graph-based method to integrate single-cell chromatin accessibility with gene expression data to infer the pseudotime ordering of cells

**DOI:** 10.1093/bioadv/vbag313

**Published:** 2026-10-15

**Authors:** Ameya Gokhale, Pawel F Przytycki

**Affiliations:** Faculty of Computing & Data Sciences, Boston University, Boston, MA 02215, United States; Faculty of Computing & Data Sciences, Boston University, Boston, MA 02215, United States

## Abstract

**Motivation:**

Pseudotime ordering methods computationally arrange individually sequenced cells along a continuous trajectory to infer their progression through biological processes. Chromatin accessibility at regulatory elements is pivotal for orchestrating the timing of gene expression during cellular differentiation. However, data sparsity and discrete grouping imposed by clustering-based approaches, often obscures subtle cell-state transitions and intermediate populations.

**Results:**

Here, we introduce Cis-TERN (Cis-Temporally Encoded Regulation Network), a graph-based method that integrates scRNA-seq and scATAC-seq to infer a pseudotime ordering of cells. Exploiting the fact that distal *cis* chromatin accessibility often precedes gene activation, Cis-TERN builds a directed bipartite graph between scRNA-seq and scATAC-seq cells with edge weights determined by either proximal or distal accessibility. Through network diffusion, Cis-TERN assigns continuous pseudotime scores to all cells. Cis-TERN infers cellular pseudotime without the need for prior biological knowledge, such as specifying a root cell type, thereby enabling unbiased reconstruction of differentiation from data alone. We show that our scores align with known lineages in healthy and tumor-derived hematopoietic cells. Additionally, Cis-TERN is able to trace individual cancer cells back to progenitor cell types. Our approach establishes a framework for leveraging chromatin accessibility and gene expression jointly to learn the timing of cell state progression.

**Availability and implementation:**

Cis-TERN is available at https://github.com/pawelab/Cis-TERN.

## 1 Introduction

Chromatin accessibility at regulatory elements plays a major role in dynamic control of gene expression determining both its timing and specificity (Corces *et al.* 2016, Klemm *et al*. 2019). By selectively allowing for transcription factors to bind to enhancer and promoter regions, cells launch the transcriptional programs suited to their development (Lara-Astiaso *et al*. 2014, Klemm *et al*. 2019). Changes in accessibility drive cellular differentiation (Buenrostro *et al.* 2013, Lara-Astiaso *et al*. 2014, Corces *et al.* 2016). In diseases such as cancer, tumors often exhibit abnormal chromatin accessibility patterns resulting in irregular differentiation (Buenrostro *et al.* 2013, Corces *et al*. 2018). Understanding chromatin accessibility and its relation to gene expression is therefore essential for determining the mechanism behind the progression of normal healthy cells into diseased ones. While chromatin accessibility proximal to and overlapping genes is generally required for them to be expressed and is therefore thought to be concurrent with a gene being expressed, distal accessibility at regulatory elements indirectly modulates the magnitude of expression (Lara-Astiaso *et al*. 2014, Buenrostro *et al*. 2015, Corces *et al.* 2016, Klemm *et al*. 2019, Granja *et al*. 2021). Crucially, distal peak accessibility often precedes gene expression and therefore can be a determinant of lineage selection (Lara-Astiaso *et al*. 2014, Ma *et al*. 2020, Li *et al*. 2023).

Advances in high-throughput sequencing technology have allowed for the measurement of chromatin accessibility (scATAC-seq) and gene expression (scRNA-seq) profiles in individual cells (Buenrostro *et al*. 2015, Corces *et al.* 2016). The resulting data has allowed researchers to better understand cell differentiation in a computationally tractable manner called pseudotime ordering where individual cells are ordered across differentiation (Trapnell *et al*. 2014, Heumos *et al*. 2023). However, because datasets generated from these techniques, especially scATAC-seq, suffer from sparsity issues, cells are typically aggregated into discrete clusters for analysis, which are interpreted as cell types or states (Heumos *et al*. 2023). Pseudotime inference typically models cellular differentiation as a sequence of transitions between these clusters or alternative lower dimensionality aggregations such as trajectories (Heumos *et al*. 2023). For example, Monocle 3, a commonly used graph-based method for pseudotime inference from scRNA-seq data, first builds a clustered graph and then fits a principal curve through those cluster partitions, assigning pseudotime first as the geodesic distance from a user-chosen root cell or cluster without further explicit consideration of each individual cell (Trapnell *et al*. 2014, Cao *et al*. 2019). Some pseudotime methods incorporate chromatin accessibility, but either treat it in isolation rather than integrating it with transcriptomics or restrict attention to proximal sites to convert accessibility into gene scores for direct integration with scRNA-seq data (Zhang *et al*., 2022, Li *et al*. 2025). These methods do not explicitly take advantage of the orthogonal information provided by chromatin accessibility. Additionally, most pseudotime inference methods do not infer directionality and require manual specification of the cell type of origin (Heumos *et al*. 2023). A related class of methods, RNA velocity, infers directionality from the ratio of unspliced to spliced transcripts within transcriptomic data (La Manno *et al*. 2018). While velocity-based approaches capture transcriptional dynamics at the level of individual cells, they require accurate spliced/unspliced count quantification and must be aggregated into a trajectory in a low-dimensional embedding. Some methods, such as MultiVelo, additionally integrate chromatin accessibility to improve inference, but rely on multiomic measurements of chromatin and expression in the same cell (Li *et al*. 2023).

To address these shortcomings, we introduce Cis-TERN (Cis-Termporally Encoded Regulation Network), a graph-based method to infer timing of differentiation in scRNA-seq data through integration with scATAC-seq data. Cis-TERN is designed to exploit the observation that distal *cis* chromatin is often accessible before a target gene is expressed. To do this, Cis-TERN constructs a bipartite graph between scRNA-seq and scATAC-seq cells with directed edges encoding either proximal or distal accessibility-based similarity between cells. Through network diffusion, it then assigns a continuous pseudotime score to all cells. We validated Cis-TERN on scRNA-seq and scATAC-seq datasets from healthy hematopoietic cells, demonstrating that Cis-TERN orders cells in a manner, i.e. consistent with known biology and additionally found that Cis-TERN is able to order cancer cells per-patient in mixed-phenotype acute leukemia (MPAL) samples (Granja *et al*. 2019). Finally, the Cis-TERN graph can be used to trace individual cells back to progenitor cell types. We applied this “reverse tracing” to terminal blood cell types to trace them back to hematopoietic stem cells (HSCs) and to cancer cells in the MPAL data to trace their progression from healthy cells.

## 2 Results

### 2.1 Overview of method

Cis-TERN is a graph-based method that generates per-cell pseudotime scores by constructing a bipartite graph whose nodes are scATAC-seq and scRNA-seq cells. Nodes are connected by weighted, directed edges. Edges from scRNA-seq cells to scATAC-seq cells encode cross modal similarity, computed as the cosine similarity between the scRNA-seq cell’s gene expression profile and the scATAC-seq cell’s proximal and gene-body accessibility (Fig. 1, grey edges). Edges from scATAC-seq cells to scRNA-seq cells are weighted by a precedence score (Fig. 1, green edges), derived from the correlation between distal *cis* peak accessibility and gene expression, reflecting the expectation that accessibility at *cis* regulatory elements often precedes transcription. For each such edge, the precedence score combines (i) the scATAC-seq cell’s chromatin accessibility, (ii) the scRNA-seq cell’s gene expression levels, and (iii) inferred peak-gene regulatory links (see Section 4.1 for details). Cis-TERN does not require matched cells between scRNA-seq and scATAC-seq modalities. The bipartite graph captures statistical relationships at the population level—cells with similar regulatory programs connect through high-scoring edges regardless of whether they originate from the same individual cell, analogous to cross-modal integration approaches that recover biological relationships from unmatched modalities by leveraging shared population structure (Granja *et al*. 2021, Hao *et al*. 2021).

**Figure 1:**
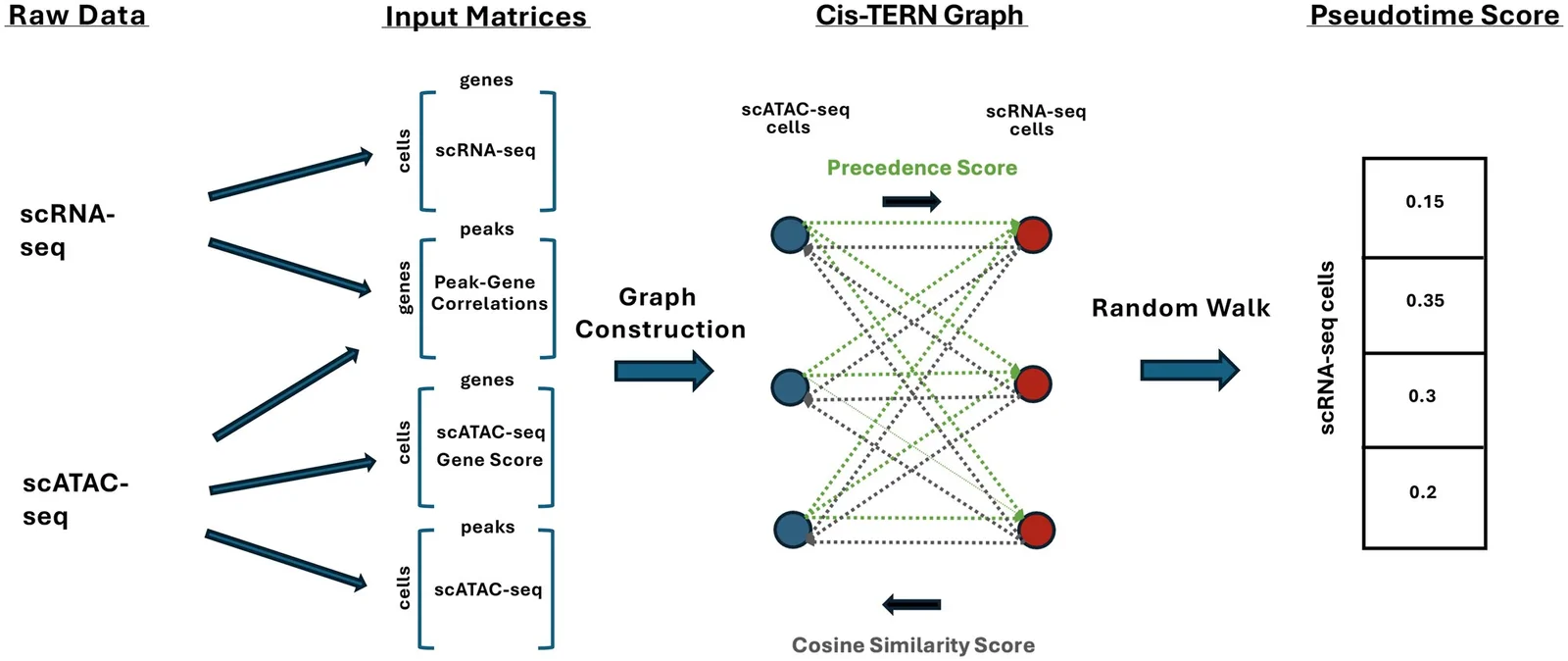
Overview of Cis-TERN. Cis-TERN takes as input four matrices generated from scRNA-seq and scATAC-seq data to construct a directed bipartite graph with edges weighted by cosine similarity in one direction and precedence score in the other on which it performs a random walk to calculate pseudotime scores for each scRNA-seq cell.

Given the sparsity and noisiness of single-cell data, individual edges will carry limited timing information. To aggregate the timing signal globally and smooth over noise, Cis-TERN performs a random walk with restart on the bipartite graph and uses the stationary probability of each scRNA-seq node as its pseudotime score. Intuitively, even weak precedence signals accumulate over long walks, biasing the stationary distribution toward later (higher-pseudotime) cells (see Section 4.2 for details).

Cis-TERN also enables “reverse tracing” individual cells to their progenitors. To do so, Cis-TERN reverses the precedence edge weights in the graph and again runs a random walk to produce a reverse pseudotime score per scRNA-seq cell, where higher values indicate cells more likely to appear earlier in pseudotime. Cis-TERN then constructs a second graph over scRNA-seq cells alone, with directed edges weighted by cosine similarity of gene-expression profiles. It then retains, for each cell, only its highest scoring connections stratified by reverse pseudotime (Supplementary Appendix Fig. 1). A Monte Carlo path sampling procedure outputs the most probable differentiation trajectory for any chosen scRNA-seq cell (see Section 4.3 for details).

### 2.2 Cis-TERN recapitulates known lineages in hematopoiesis

We tested Cis-TERN’s functionality on scRNA-seq (35 882 cells) and scATAC-seq (36 690 cells) data from healthy bone marrow and peripheral-blood mononuclear cells (PBMCs) and six samples of mixed phenotype acute leukemia (MPAL) consisting of a total of 18 056 scRNA-seq cells and 35 423 scATAC-seq cells also collected from bone marrow and peripheral blood (Granja *et al*. 2019).

To assess Cis-TERN’s ability to assign pseudotime scores to individual cells, we first tested whether it recapitulates established differentiation lineages in the scRNA-seq and scATAC-seq cells from the healthy individuals. The scRNA-seq had previously been clustered into 26 hematopoietic states including lineages for monocytes and B cells. We ran Cis-TERN on the full combined scRNA-seq and scATAC-seq data to infer pseudotime scores for all cells in both lineages (Lara-Astiaso *et al*. 2014, Corces *et al.* 2016). As expected, Cis-TERN scores increase monotonically on average across successive cell types in both known lineages (Fig. 2a for monocytes, Fig. 2b for B cells, and Supplementary Appendix Fig. 2 for all cell types) with the exception of HSCs, which have an inflated number of incoming non-zero edges (Supplementary Appendix Fig. 3) due to an excess of weak peak-gene links resulting in increased precedence scores (Supplementary Appendix Fig. 4).

**Figure 2:**
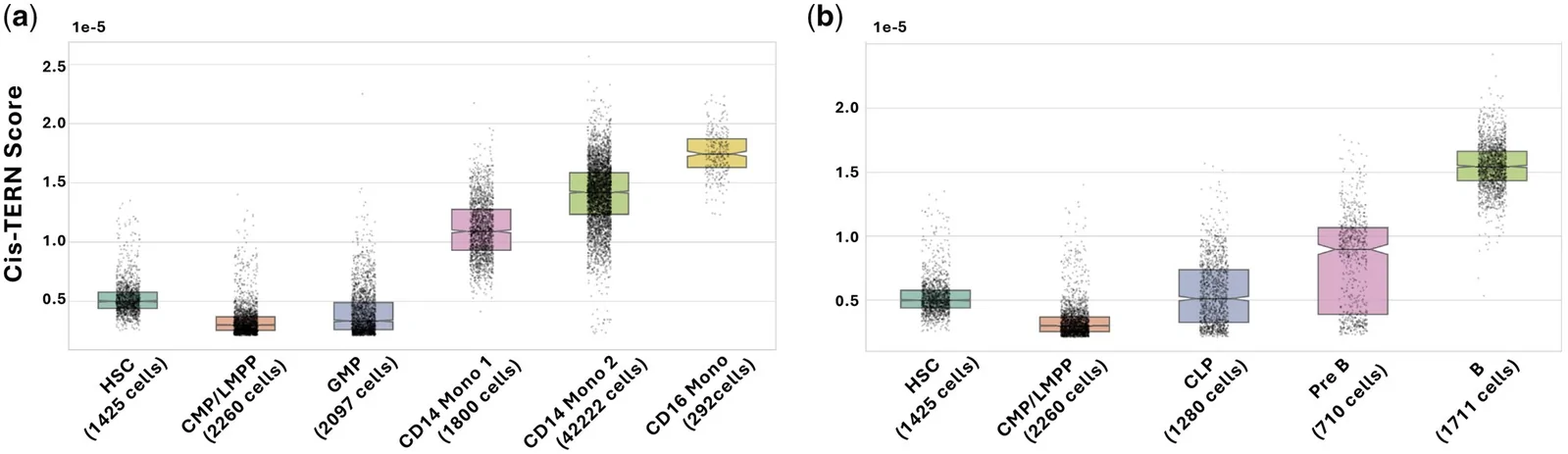
Cis-TERN orders cells from known lineages in hematopoiesis. Notched boxplots of the Cis-TERN pseudotime scores for cells annotated to cell types ordered by their lineages for (a) monocytes and (b) B cells.

**Figure 3:**
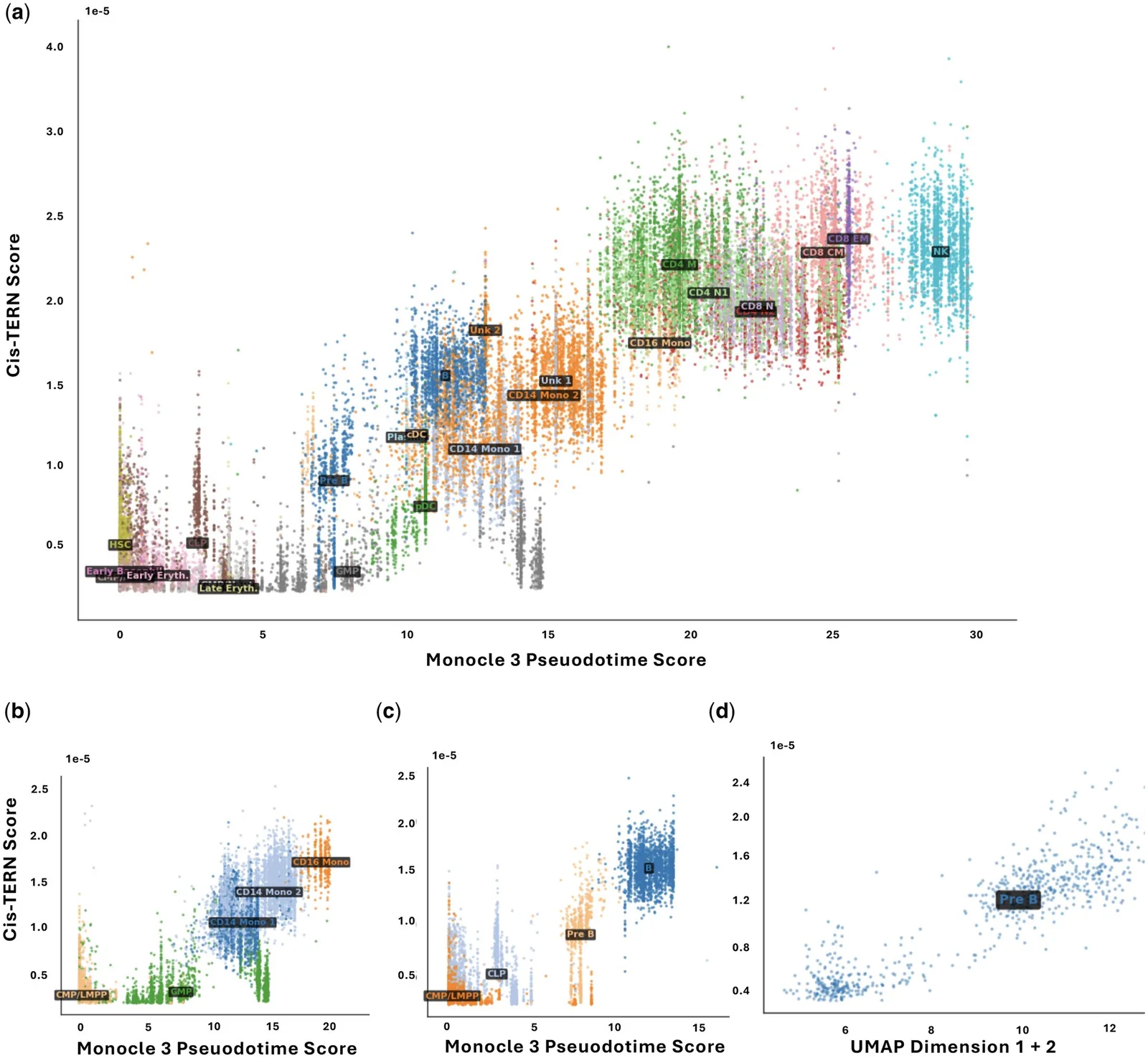
Cis-TERN captures the continuous progression of cells. Cis-TERN scores are highly correlated to Monocle 3 pseudotime scores across (a) all scRNA-seq cells (b) the monocyte Lineage and (c) the B-cell lineage. (d) Cis-TERN scores for pre-B cells are highly correlated with the UMAP embedding of scRNA-seq PBMC data (Granja *et al*. 2019). In all panels, cells are colored based on annotated cell type labels and cell type labels appear at the centroid of all cells assigned to that cell type.

**Figure 4:**
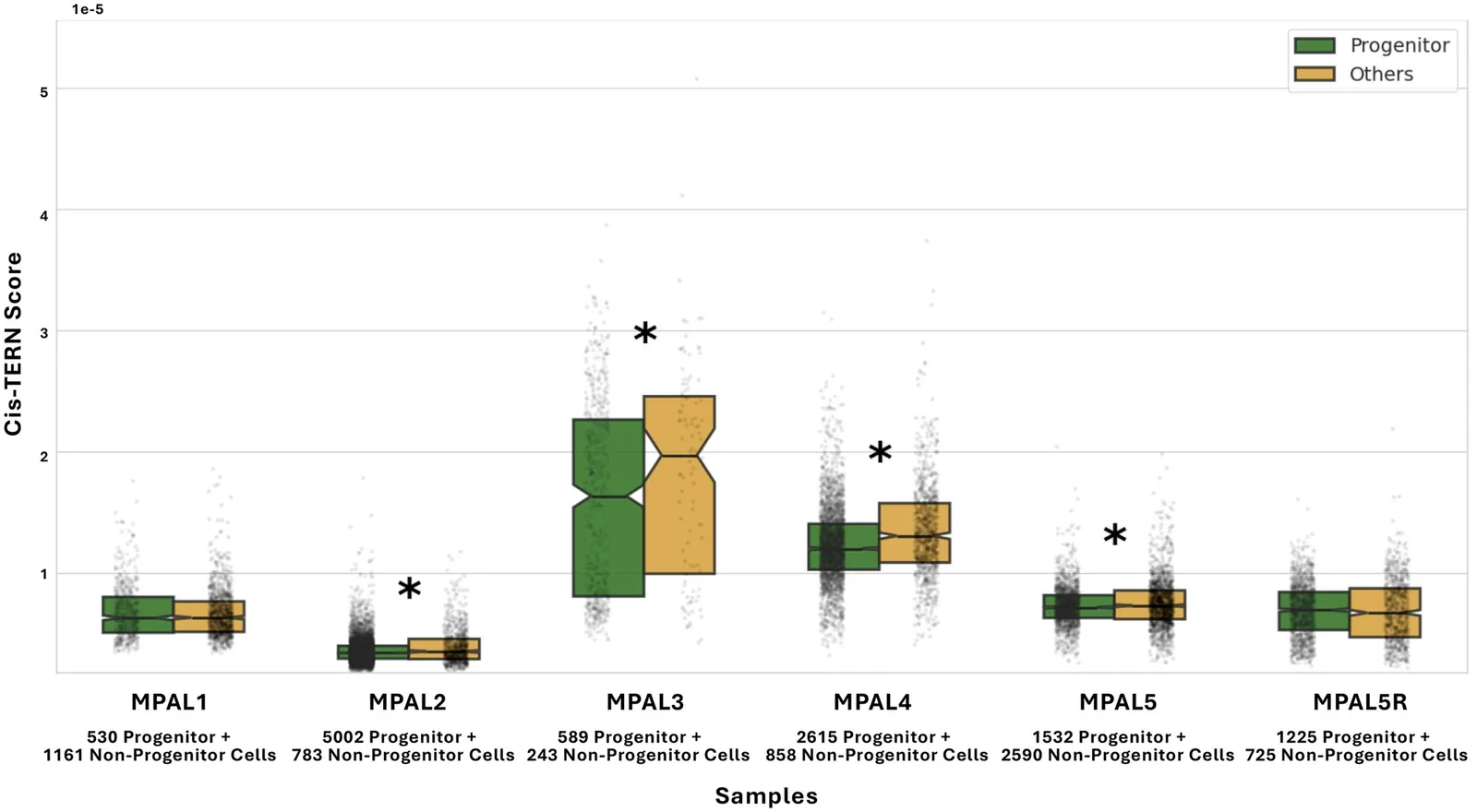
Cis-TERN differentiates progenitor-like cancer cells. For four of six tumor samples, progenitor-like cells (green notched boxplots) had significantly lower Cis-TERN scores than other cancer cells (orange notched boxplots). Significance level of a one-tailed Wilcoxon test of *P*-value < .05 is indicated with a *.

Next, we examined how Cis-TERN captures the continuous progression of cells both across cell types and within cell types. Since a ground truth is not known, we compared Cis-TERN’s pseudotime scores to those of Monocle 3 (Trapnell *et al*. 2014), and scDART (Zhang *et al*. 2022), and to the ordering captured by a low dimensional UMAP embedding, which can capture local cell type similarity and progression, including in hematopoiesis (La Manno *et al*. 2018, Hao *et al*. 2024). When running Monocle 3 on integrated scRNA-seq and scATAC-seq data with HSCs set as the root, we observed that, across all cells, Cis-TERN’s pseudotime scores correlate well those generated by Monocle 3 despite Cis-TERN having no prior knowledge of a cell type of origin (Fig. 3a, Pearson’s *⍴* = 0.89). We similarly observed that within the monocyte and B-cell lineages, the two models broadly agreed (Fig. 3b the for monocyte lineage, Pearson’s *⍴* = 0.77 and Fig. 3c for the B-cell lineage, Pearson’s *⍴* = 0.85). In both lineages, Cis-TERN showed a better ability to assign a broad range of scores to cells within the same annotated cell type and generated smoother, less discretized transitions between cell types. Cis-TERN captures continuous transitions at key developmental boundaries that appear discrete under clustering based pseudotime. At the CMP/LMPP to GMP and CLP to Pre-B transitions, Cis-TERN shows substantially lower silhouette scores (0.057 and 0.186 respectively) compared to Monocle 3 (0.581 and 0.729), indicating that Cis-TERN resolves these transitions as gradients rather than discrete states. We further compared Cis-TERN to scDART (Zhang *et al*. 2022), a deep learning method that integrates unmatched scRNA-seq and scATAC-seq to learn a shared latent space for trajectory inference. With HSCs specified as the root, scDART produces pseudotime orderings initially consistent with known hematopoietic biology but shows difficulty ordering terminal cell types in the myeloid lineage (cell-type level Kendall’s tau = 0.87), whereas Cis-TERN correctly places these populations only struggling with HSCs (Kendall’s tau = 0.87, Supplementary Appendix Fig. 5). A key advantage of Cis-TERN over both scDART and Monocle 3 is that it does not require specification of a root cell to establish directionality, nor does it rely on clustering cells into discrete cell types.

**Figure 5:**
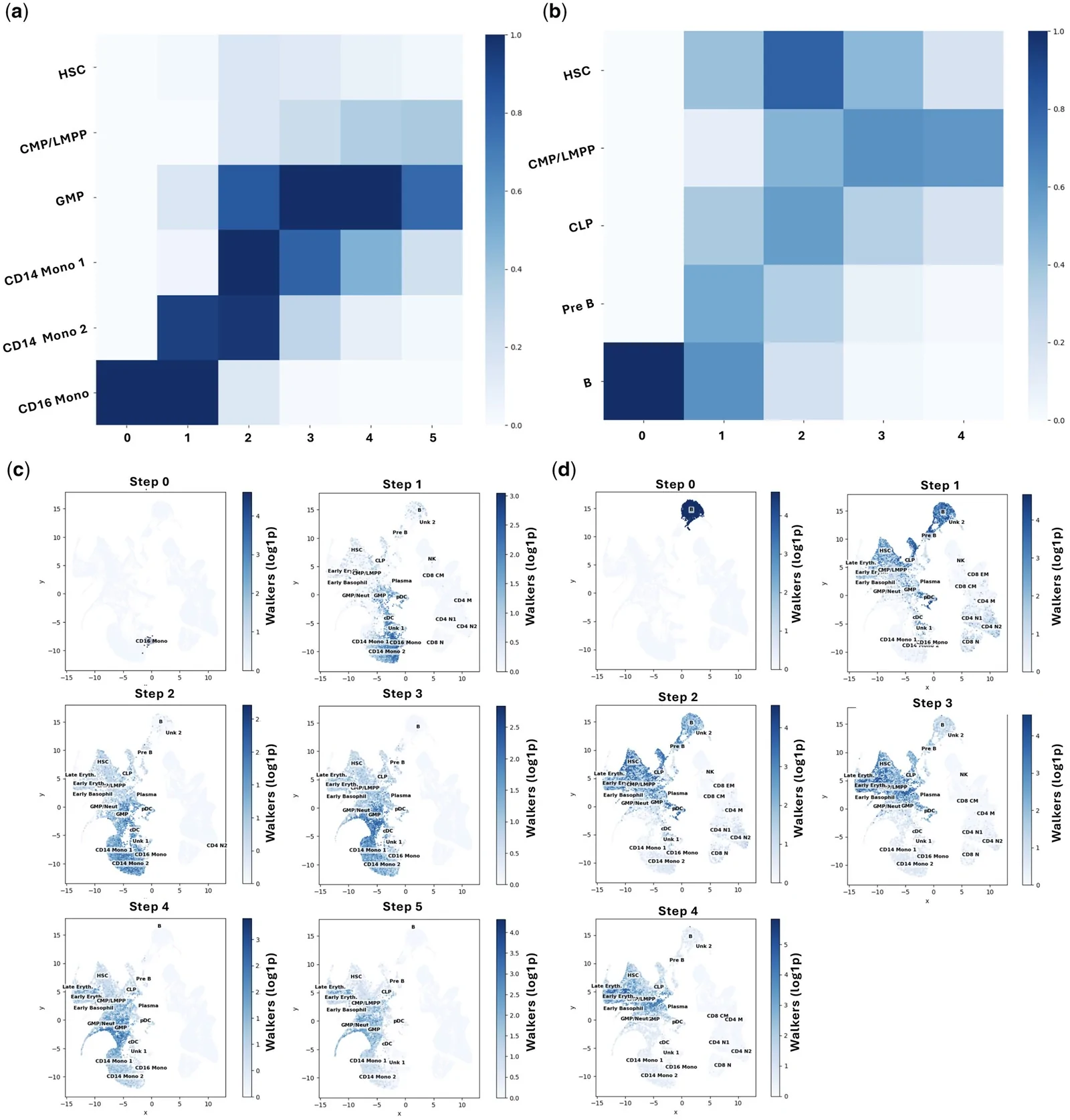
Reverse tracing terminal cell types to progenitors. The min-max scaled fraction of traces that visited each cell type in (a) the monocyte and (b) the B-cell lineage when all traces starting at the corresponding terminal cell type are aggregated. The number of individual traces starting at the terminal cells for (c) the monocyte and (d) the B-cell lineage observed to be at every cell in the UMAP embedding of the scRNA-seq data at each time step (Granja *et al*. 2019).

To further evaluate the local ordering of our scores, we compared them with a UMAP embedding of the scRNA-seq data (Supplementary Appendix Fig. 6a) (Becht *et al*. 2018, Granja *et al*. 2019). We observed that Cis-TERN’s pseudotime scores correlate well with the embedding across both lineages (Supplementary Appendix Fig. 6b, Pearson’s *⍴* = 0.60 for the monocyte lineage and Supplementary Appendix Fig. 6c, Pearson’s *⍴* = 0.81 for the B-cell lineage). We similarly found that, within each cell type, our scores correlated with the embedding, including for transitionary cell types such as pre-B cells (Fig. 3d, Pearson’s *⍴* = 0.90) and for cell types that appear more continuous than discrete such as CD14 monocytes types 1 and 2 (Supplementary Appendix Fig. 6d, Pearson’s *⍴* = 0.49). Additionally, while, as observed above, the median HSC Cis-TERN score was not the lowest among all cell types, the local gradient is in agreement with the embedding highlighting our method’s ability to resolve progressive differentiation within the population (Supplementary Appendix Fig. 6e, Pearson’s *⍴* = 0.27).

**Figure 6:**
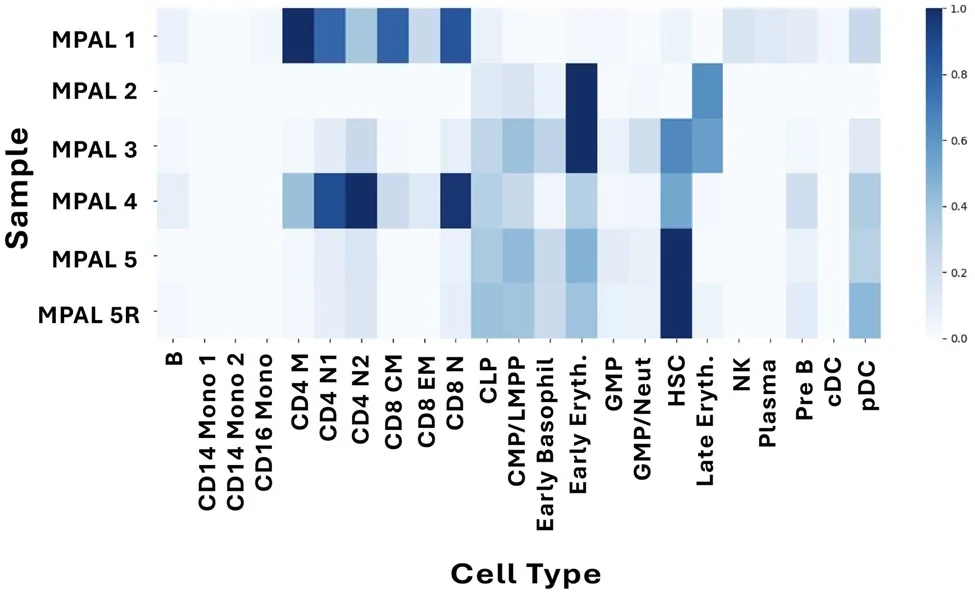
Reverse tracing cancer cells reveals tumor heterogeneity. The fraction of trace that ends at each cell type after three steps when starting at cancer cells in the given sample.

### 2.3 Cis-TERN is efficient and robust

In addition to hematopoiesis, we found that Cis-TERN is able to order cell types in the developing brain, an organ with complex lineages (Nowakowski *et al*. 2017, Trevino *et al*. 2021) (Supplementary Appendix Fig. 7), and in an in vitro cardiac differentiation time course from iPSCs to mature cardiomyocytes (Ameen *et al*. 2022), correctly ordering cell types along the known trajectory (Supplementary Appendix Fig. 8a) and samples by timepoint (Supplementary Appendix Fig. 8b). To quantitatively benchmark this dataset, we computed sample-level (per day) Kendall’s tau against the order of sampled timepoints for Cis-TERN, Monocle 3, and scDART. Cis-TERN and scDART both achieved a Kendall’s tau of 0.8. Monocle 3, in contrast, was unable to reconstruct a single connected trajectory across the full time course, producing a disjoint graph whose resulting ordering depended on the choice of the root cell. We further compared Cis-TERN to MultiVelo (Li *et al*. 2023), a method that jointly models RNA velocity and chromatin accessibility dynamics from multiome measurements, on a 10× Genomics developing mouse brain dataset (Genomics 2021), observing a correlation between Cis-TERN pseudotime scores and MultiVelo latent time (Pearson’s *⍴* = 0.47). We note that Cis-TERN achieved this while using scATAC-seq and scRNA-seq independently rather than as matched multiome measurements. Like velocities, Cis-TERN scores can also be embedded into UMAP space producing a vector field showing the direction of differentiation for each cell (Supplementary Appendix Fig. 9). We additionally compared Cis-TERN to scDART and Monocle 3 on this brain dataset, computing cell-type-level Kendall’s tau against the known differentiation trajectories for the radial glia and glutamatergic neuron lineages. Both Cis-TERN and scDART correctly ordered the shorter radial glia lineage (Kendall’s tau = 1.0 for both), while Monocle 3 failed to do so (Kendall’s tau = 0.33). On the longer and more complex glutamatergic neuron lineage, Cis-TERN retained a strong ordering (Kendall’s tau = 0.62), while both scDART and Monocle 3’s orderings were substantially misordered (Kendall’s tau = -0.52 and 0.24 respectively). Overall, this indicates that Cis-TERN is able to recapitulate the broad global ordering of cell lineages with no prior knowledge.

Given that scATAC-seq data is typically less readily available than scRNA-seq data (Chen *et al*. 2019), we next examined robustness of Cis-TERN to smaller quantities of scATAC-seq cells. We ran Cis-TERN on the full PBMC scRNA-seq dataset described above while holding out all but one scATAC-seq sample at a time. This captures the common experimental practice of performing scRNA-seq on all samples but scATAC-seq on only a subset (Chen *et al*. 2019). In this setting, Cis-TERN recapitulated lineage progression in both B cells and monocytes trajectories with as around 10 000 scATAC-seq cells. At substantially lower coverage (i.e. fewer than 5000 scATAC-seq cells), performance degraded, suggesting a practical lower bound on input size for reliable reconstruction (Supplementary Appendix Fig. 10). Across these samples, Cis-TERN completed scoring in around 45 seconds for samples with around 3000 cells to just over 2 minutes for the full dataset with over 35 000 cells per modality, indicating favorable runtime scaling, with memory scaling linearly with dataset size (Supplementary Appendix Fig. 11).

As an additional robustness test, we examined the performance of Cis-TERN using precedence scores or cosine similarities alone. To evaluate precedence scores, we took their sum per scRNA-seq cell and observed that they were able to partially order the lineages alone (Supplementary Appendix Fig. 12a and b) and were highly correlated with Cis-TERN scores per cell type (Supplementary Appendix Fig. 12c). To evaluate cosine similarity alone, we replaced precedence weights with cosine similarity in the bipartite which resulted in complete loss of ordering of cell types in lineages as expected (Supplementary Appendix Fig. 12d and e). To quantify the relative contributions of each component, we computed Kendall’s tau between each method’s score and the known lineage ordering. For the monocyte lineage, Kendall’s tau was 0.58 for the full Cis-TERN model, 0.52 for precedence alone and 0.08 for cosine similarity. For the B-cell lineage, values were 0.46, 0.44, and −0.05 respectively. While precedence scores alone achieve substantial ordering, the full framework additionally smooths noisy single-cell measurements as measured by the coefficient of variation across cells within each cell type which is substantially reduced in the full model compared to precedence sums alone (Supplementary Appendix Fig. 13), confirming that cosine similarity anchoring helps filter noisy precedence edges by routing flow through biologically matched ATAC cells. A perturbation analysis removing all peak-gene links to an increasing fraction of genes shows gradual monotonic degradation of lineage ordering with no sharp decline, indicating that Cis-TERN does not depend critically on a small number of regulatory annotations (Supplementary Appendix Fig. 14). Finally, to validate Cis-TERN’s graph construction we examined cosine similarity and precedence scores for matched RNA-ATAC cell pairs in Mouse Brain E18 multiome data (Genomics 2021). As expected, we observe that the matching cell is typically among the highest cosine similarity edges but not the precedence score edges, consistent with the notion that simultaneous measurements reflect high cross-modal similarity but minimal temporal offset (Supplementary Appendix Fig. 15).

### 2.4 Cis-TERN orders cancer cells from MPAL samples in a per patient manner

Finally, we used Cis-TERN to pseudotime order cancer cells from MPAL tumor samples in a per patient manner. The six MPAL samples were classified as T-myeloid MPAL (samples “MPAL1”—“MPAL3”), B-myeloid MPAL (sample “MPAL4”), T-myeloid MPAL sampled before CALGB chemotherapy (sample “MPAL5”), and post-treatment relapse (sample “MPAL5R”) (Granja *et al*. 2019). To score each sample, we ran Cis-TERN on the combined full healthy hematopoiesis data with the scRNA-seq and scATAC-seq data for that sample (see Section 4.4 for details). First, we observed that, as expected (Zeng *et al*. 2025), the ordering of the healthy cells was largely unchanged by adding the tumor data (Supplementary Appendix Fig. 16), consistent with the established practice of treating healthy hematopoiesis as a stable reference with which blood cancers are projected (Granja *et al*. 2021, Zeng *et al*. 2025), and that the ordering is robust to increasing cancer cell proportions (Supplementary Appendix Fig. 17). Across the tumor samples, while there is no ground truth for the time ordering of cancer cells, we observed that cells that had been annotated as “progenitor like” had earlier Cis-TERN pseudotime scores (Fig. 4). This indicates that Cis-TERN was broadly able to, in individual samples, sort cancer cells by their plasticity.

### 2.5 Cis-TERN orders cancer cells from MPAL samples in a per patient manner

In order to evaluate Cis-TERN’s capacity to “reverse trace” individual cells to their progenitors, we ran Cis-TERN’s reverse tracing procedure on the terminal cell types in healthy hematopoiesis. In particular we ran it on all 256 CD16 monocytes (the terminal cell type of the monocyte lineage) and on all 1711 B cells (the terminal cell type of the B-cell lineage). For each terminal type, we then aggregated the traces from all the cells and observed that the majority of traces moved back through the lineage to progenitor cell types (Fig. 5a for CD16 monocytes and Fig. 5b for B cells) though the traces do make additional spurious visits to other cell types as would be expected to occur due to stochastic variability (Supplementary Appendix Fig. 18). The number of steps taken before termination is automatically determined as described in Section 4.3 (see Supplementary Appendix Fig. 19 for calculated distribution of walk lengths). To explore global per-cell reverse tracing dynamics, we used the precomputed UMAP of scRNA-seq cells to observe the cells visited by the traces in both lineages (Fig. 5c for CD16 monocytes and Fig. 5d for B cells). As expected, the density of the traces moves gradually towards progenitor cell types.

Finally, given Cis-TERN’s ability to retrace the paths of healthy terminal state cells, we tested how Cis-TERN would reverse trace cancer cells to possible healthy progenitors. We applied the reverse tracing procedure to the per-sample integrations described in Section 2.2 aggregating the traces from all cancer cells in each sample and observed that cancer cells had sample specific origins (Fig. 6) to which traces converge to after a small number of steps (Supplementary Appendix Fig. 20). Cancer cells from two samples (MPAL1 and MPAL4) traced primarily back to T cells, while two samples (MPAL2 and MPAL3) had similarity to erythroid cells, as was previously observed (Granja *et al*. 2019). Finally, MPAL5 and MPAL5R, which were taken from the same individual prior to chemotherapy and after treatment and relapse, showed very similar cancer origins as would be expected and was previously observed (Granja *et al*. 2019). To independently validate these origins, we examined CITE-seq protein measurements for the same cancer cells. MPAL1 and MPAL4, which Cis-TERN traces to T-cell progenitors, show significant enrichment for T-cell surface markers CD3 (3.35-fold and 7.42-fold respectively) and CD8A (3.87-fold and 2.19-fold respectively) compared to other MPAL samples (one-tailed Wilcoxon *P*-value <.0001 for both comparisons), providing orthogonal experimental support for Cis-TERN’s reverse tracing results.

## 3 Discussion

Cis-TERN provides a framework for integrating scRNA-seq and scATAC-seq measurements to infer cellular differentiation by leveraging the precedence of distal chromatin accessibility. Our approach enables the reconstruction of developmental progressions without prior knowledge of root cell states and successfully aligns with established biological lineages in healthy hematopoiesis. Additionally, Cis-TERN retains favorable scaling to tens of thousands of cells per modality. Beyond forward ordering, Cis-TERN’s unique reverse tracing allows individual cells to be mapped back to their putative progenitors. We demonstrate this in the context of mixed-phenotype acute leukemia where Cis-TERN mapped malignant cells to which healthy precursors they likely differentiated from.

While RNA velocity-based methods offer a related approach to inferring directionality; they require spliced/unspliced count quantification which can be noisy, and methods that additionally integrate chromatin accessibility require matched multiome measurements—data modalities that remain scarce relative to independently collected scRNA-seq and scATAC-seq. Cis-TERN instead leverages temporal lag between distal chromatin accessibility and gene expression, requiring only independently collected data from each modality. Unlike methods that operate on scRNA-seq alone, Cis-TERN derives its directional signal from the temporal relationship between distal chromatin accessibility and gene expression—a signal absent in transcriptomic data alone and one that does not require cells to be clustered into discrete types or a root cell type to be manually specified. As observed in the iPSC-to-cardiomyote differentiation timecourse where root choice produced distinct partial trajectories in Monocle 3, removing the need for root specification can help create consistent pseudotime orderings of all cells in a study.

A central assumption of Cis-TERN is that distal chromatin accessibility tends to precede activation of its target genes. While well-supported in developmental contexts such as hematopoiesis (Lara-Astiaso *et al*. 2014, Li *et al*. 2023), this assumption has not been exhaustively validated across all cell-state transitions. In particular, in acute stimulus or perturbation responses, many genes are activated through pre-existing primed enhancers that were already accessible prior to stimulation (Alasoo *et al*. 2018, Maytum *et al*. 2024), effectively collapsing the temporal gap that Cis-TERN relies on. Cis-TERN is therefore best suited to transitions involving substantive activation of new regulatory programs. Similarly, cells with broadly accessible chromatin states such as iPSCs may require stricter peak-gene correlation thresholds to isolate lineage-specific regulatory signals from global accessibility patterns.

A second class of limitations concerns the topology of the underlying biological process. Cis-TERN produces a single scalar pseudotime per cell from the stationary distribution of a random walk, which is intrinsically a one-dimensional ordering. In bifurcating or multi-branch lineages, cells at comparable developmental stages on different branches will tend to receive similar forward scores, and distinct fates are not resolved by the forward score alone. The reverse tracing procedure partially compensates for this by producing per-cell, branch-specific progenitor paths through the cosine-similarity graph, as demonstrated by the lineage-appropriate traces from CD16 monocytes and B cells despite both converging on HSCs. A second limitation concerns cyclic processes—Cis-TERN’s directional signal derives from the asymmetry of distal accessibility preceding gene activation, which has no analogue in cell-cycle progression where accessibility and expression changes are periodic. Cells with strong cell-cycle activity rather than developmental dynamics, consistent with the HSC inflation we observe in healthy hematopoiesis, which we attribute to self-renewal.

An additional limitation is that lineage ordering within cancer cells themselves may not be faithfully respected. For example, MPAL cells often display lineage-promiscuous and stem-like transcriptional profiles that do not map cleanly onto a single canonical differentiation trajectory (Mumme *et al*. 2025). We therefore can only interpret cancer-cell Cis-TERN scores with respect to the healthy hematopoietic backbone, asking which healthy progenitor state each cancer cell most resembles in its developmental position.

A core component of Cis-TERN are the peak-gene link predictions used to find distal *cis* regulatory elements to associate with each gene for calculating precedence scores. The current implementation of Cis-TERN uses across-cell-type correlation between scRNA-seq and scATAC-sec data as an indication of linkage. However, this is a coarse-grained view that only captures the subset of links, i.e. present in common cell types. In the future, the integration of additional data modalities such as H3K27ac and Hi-C could enhance link prediction to generate better precedence scores by identifying active enhancers and enhancer-promoter contacts.

Overall, Cis-TERN is an immensely flexible framework for integrating scATAC-seq and scRNA-seq data. The encoding of this multimodal data into a weighted bipartite graph leaves room for alternative edge weights and scales well to large quantities of single-cell sequencing data.

## 4 Methods

Cis-TERN generates a pseudotime ordering of cells by integrating scRNA-seq and scATAC-seq data by building a graph (described in Section 4.1) and then walking on the graph to score cells (described in Section 4.2). It can trace cells back to their origins (described in Section 4.3). Datasets used, data processing, and details of how the method was run are described in Section 4.4. Code availability is described in Section 4.5.

### 4.1 Generating Cis-TERN graph

As input, Cis-TERN takes four matrices. *S_G_* is a cell-by-gene matrix of scRNA-seq gene expression counts, depth-normalized to 10 000 counts per cell. *S_A_* is a cell-by-peak binary accessibility matrix from scATAC-seq, where each entry indicates whether a chromatin peak is accessible in a given cell. *S_M_* is a cell-by-gene score matrix derived from scATAC-seq data, where each entry quantifies the proximal and gene body chromatin accessibility of a given gene in a given cell, depth-normalized to 10 000 counts per cell. This matrix serves as an accessibility-based proxy for gene expression within the scATAC-seq modality. *S_L_* is a gene-by-peak correlation matrix where each entry represents the Pearson correlation between a gene’s expression across cell types (from scRNA-seq) and a peak’s accessibility across cells (from scATAC-seq), restricted to peaks within 250 kb of the gene’s TSS; this matrix encodes putative distal *cis*-regulatory relationships between peaks and genes (Chen *et al*. 2019).

These matrices can be generated from one or more scATAC-seq and scRNA-seq experiments following the data processing steps described in Section 4.4. Note that multiome data is not required and thus the cells in *S_G_* do not need to be the same as the cells in *S_A_*. Using these matrices, Cis-TERN generates a directed bipartite graph where nodes are scATAC-seq and scRNA-seq cells. There are two types of directed edges between each group of cells as follows:

Precedence Score Edges: These are directed edges from scATAC-seq to scRNA-seq cells. A precedence score between an scATAC-seq cell and scRNA-seq quantifies how strongly the scATAC-cell precedes the scRNA-seq cell based on distal accessibility of peaks *cis* to expressed genes. We calculate the cell-by-cell matrix of precedence scores *P* in the following manner:


(1) $$P=S_{A}{\left(S_{G}S_{L}\right)}^{T}$$

Some values in *P* will be negative due to negative correlations between peaks and genes. These values are set to 0, as the precedence matrix serves as edge weights in a Markov chain, requiring non-negative entries to define valid transition probabilities.

Cosine Similarity Score Edges: These are directed edges from scRNA-seq to scATAC-seq cells. A cosine similarity score captures how similar each scRNA-seq cell’s gene expression (*S_G_*) is to each scATAC-seq cell’s proximal and gene body accessibility profile (*S_M_*), computed as the cosine similarity between the two vectors in gene space. *S_M_* serves as an accessibility-based proxy for gene expression in the scATAC-seq modality, enabling direct comparison between modalities in a shared gene space. This results in a cell-by-cell matrix *C* where each entry *C_i, j_* is the cosine similarity of cell *i* in the scATAC-seq data and cell j in the scRNA-seq data across genes.

The final Cis-TERN graph can be written as *G = (V, E)* where *V* is all scATAC-seq cells and scRNA-seq cells and *E* is the two sets of directed precedence and cosine similarity edges. We represent this graph with the square adjacency matrix *A* where the number of rows and columns equal to the total number of cells and where the top right quadrant is *P* and bottom left quadrant is *C^T^*.

### 4.2 Cis-TERN pseudotime scoring

To capture temporal progression, Cis-TERN performs a random walk with restart. We first make the adjacency matrix *A* column stochastic. To allow for restarts, we then calculate the transition matrix *G* as follows:


$$G=\alpha A+(1-\alpha )\frac{1}{N}11^{T}$$

where *N* is the total number of nodes in the graph (scATAC-seq + scRNA-seq cells), 11^*T*^ is a matrix of all ones (used to uniformly transport a node for a restart), and α is the damping factor that controls the probability of teleportation. Adding a restart probability guarantees the existence of a unique steady state distribution which is a vector of probabilities π satisfies π = *G*π.

Cis-TERN uses an iterative method to find the steady state distribution by initializing π_0_ to be uniform and then iterating:


(3) $$\pi_{t+1}=G\pi_{t}\;\text{until}\;\|\pi_{t+1}-\pi_{t}\|<\varepsilon$$

Upon convergence, the final vector π* is an approximation of the true underlying steady state distribution. Each scRNA-seq cells value in π* is its Cis-TERN score. Intuitively, a higher score means a random walker will visit that cell more in the long run, implying a later position in pseudotime.

### 4.3 Cis-TERN reverse tracing

To trace backward from a given scRNA-seq cell and infer its putative progenitors, we modify the Cis-TERN bipartite graph by inverting the precedence weights giving a reverse precedence score *R *= 1/*P*. In the original graph, a larger precedence weight on edge A to B implies that B lies later in pseudotime than A. After inversion, this temporal signal is reversed: higher weights now favor transitions to cells that are earlier in development.

Cis-TERN then constructs an adjacency matrix A* where the top right quadrant is *R* and bottom left quadrant is *C^T^*. We run the same Cis-TERN random walk algorithm described in Section 4.2 with this new graph. In this case a higher random walk score indicates that the cell came earlier in development trajectory with the final steady state vector *t* capturing a “reverse Cis-TERN score” for the scRNA-seq cells. Cis-TERN next builds a pairwise cosine similarity matrix *S_S_* from *S_G_* only keeping the top 10% of entries by cosine similarity in each row (scRNA-seq cell) and setting the rest to 0. Additionally, for each row, Cis-TERN only keeps the edges to other cells that have a greater reverse Cis-TERN score.

Finally, to trace individual cells back to their progenitors, Cis-TERN runs a Monte Carlo simulation for up to 10 steps starting with 100 walkers on the scRNA-seq cell of interest. At each step, every walker moves according to row-normalized (row-stochastic) edge weights in *S_S_*; rows with all zeros are treated as sinks (walkers remain in that cell). To determine a principled stopping point, Cis-TERN employs an entropy-based termination rule. At each step, it computes the entropy of the walker distribution over cells and applies an elbow criterion: the optimal step is chosen as the one with the largest perpendicular distance to the chord connecting step 1 and the first step at which entropy reaches zero. Alternative stopping criteria including fixed step counts and entropy threshold rules were evaluated but found to be less robust across cell types rather than the elbow criterion, which adapts to the shape of the entropy curve for each individual trace; progenitor cell type assignments were found to be stable across steps flanking the chosen stopping point.

### 4.4 Datasets used, data processing, and method run details

#### 4.4.1 Data sources

All hematopoiesis (healthy and MPAL) scATAC-seq data was downloaded from GSE139369 as fragment files. scRNA-seq data was obtained from the study’s public GitHub repository as two separate SummarizedExperiment objects: one containing healthy samples, and one for the combined healthy and MPAL dataset. Both objects included curated cell type annotations for all cells in the healthy and MPAL compartments, as provided by the original authors.

#### 4.4.2 Preprocessing

The scRNA-seq data was depth-normalized to 10 000 counts per cell to generate the input cell-by-gene expression matrix for Cis-TERN. The scATAC-seq fragment files were processed with ArchR v1.0.2 using default parameters using the functions ‘addArchRGenome(“hg19”)’, ‘createArrowFiles’, ‘addDoubletScores’, ‘ArchRProject’, ‘filterDoublets’, ‘addIterativeLSI’, ‘addHarmony’, ‘addClusers’, ‘addUMAP’, ‘getMarkerFeatures’, ‘addImputeWeights’, ‘addGroupCoverages’, ‘addReproduciblePeakSet’, ‘addPeakMattrix’ in order. ArchR’s binarized cell-by-peak matrix was used as the input cell-by-peak matrix for Cis-TERN. ArchR’s ‘GeneScoreMatrix’ which is a proximal and gene body accessibility-based cell-by-gene matrix, i.e. by default depth-normalized to 10 000 counts per cell, was used as the input cell-by-gene gene score matrix for Cis-TERN. The gene-by-peak peak-gene correlation input matrix was generated by ArchR’s ‘addPeak2GeneLinks’ using the gene integration matrix. The integrated expression matrix was generated by pairing scATAC-seq data with scRNA-seq using ArchR’s ‘addGeneIntegrationMatrix’ function. For the ‘seRNA argument’, we supplied a SummarizedExperiment containing the scRNA-seq data, first subsetting it to include only cells from those samples that were also present in the scATAC-seq dataset, so that integration was performed exclusively between matched samples.

#### 4.4.3 Method run details

We ran Cis-TERN on the data in the following combinations: all healthy ATAC with all healthy RNA, one healthy ATAC at a time with all healthy RNA, and one MPAL ATAC and matching RNA with all healthy ATAC and RNA. In each combination, the data preprocessing is run on the given set of data and only genes in common across the two gene-by-cell matrices are kept. In all cases, Cis-TERNs random walk used a damping factor *α*  =  0.85 and a power-iteration convergence tolerance *ε*  =  1 × 10^−^^6^. To run Monocle 3, we used the downloaded healthy scRNA-seq SummarizedExperiment and scATAC-seq gene score summarized experiment (generated with ArchR “getMatrixFromProject” with option useMatrix set to “GeneScoreMatrix”). These were converted to Seurat objects using Seurat v5.0.3 function ‘CreateSeuratObject’ (with ‘counts’ set to gene score counts or expression and ‘meta.data’ set to gene score matrix or scRNA-seq SummarizedExperiment column data respectively), log-normalized, and subjected to standard processing with default parameters (highly variable gene selection, scaling and principal component analysis with nPC = 30) (Hao *et al*. 2024). We integrated the scRNA-seq and gene score datasets in gene space using Seurat’s canonical correlation analysis-based integration workflow (‘SelectIntegrationFeatures’, ‘FindIntegrationAnchors’ and ‘IntegrateData’), followed by scaling, PCA and UMAP on the integrated assay. The integrated Seurat object was converted to a Monocle 3 ‘cell_data_set.’ Trajectory learning and pseudotime estimation were performed in Monocle 3 using ‘cluster_cells’, ‘learn_graph’, and ‘order_cells’, with hematopoietic stem cells (annotation ‘01_HSC’ in the ‘BioClassification’ metadata) specified as the root population. The resulting pseudotime values were computed for all cells in the integrated project. For downstream analyses we retained only pseudotime assigned to the scRNA-seq cells.

### 4.5 Code availability

Cis-TERN is an open-source method available at https://github.com/pawelab/Cis-TERN which includes a jupyter notebook with a sample run for pseudotime scoring and reverse tracing functionality.

## Supplementary Material

vbag313_Supplementary_Data

## Data Availability

No datasets were generated during the current study. All analysed data is publicly available as described in the methods.
