# Supplementary material for: Cis-TERN: a graph-based method to integrate single-cell chromatin accessibility with gene expression data to infer the pseudotime ordering of cells
Source: Bioinform Adv. 2026 Oct 15;6(1):vbag313. doi: 10.1093/bioadv/vbag313 (PMC13721134; doi:10.1093/bioadv/vbag313)
Supplement: Supplementary file 1 [file vbag313_supplementary_data.pdf]

Appendix

The appendix contains the following additional figures:

Figure 1: Overview of reverse tracing with Cis-TERN
Figure 2: Cis-TERN scores for all cell types in healthy hematopoiesis
Figure 3: HSCs have an inflated number of incoming non-zero edges
Figure 4: Effect of peak-gene correlation threshold on Cis-TERN pseudotime ordering in hematopoiesis
Figure 5: Comparison of pseudotime ordering between Cis-TERN and scDART on healthy hematopoiesis data
Figure 6: Comparison of Cis-TERN scores to UMAP embedding
Figure 7: Cis-TERN orders cell lineages in brain development
Figure 8: Cis-TERN pseudotime scores for in vitro cardiac differentiation time course
Figure 9: Visualization of Cis-TERN as a velocity-inspired vector field.
Figure 10: Robustness of Cis-TERN to smaller quantities of scATAC-seq cells
Figure 11: Cis-TERN runtime scaling and memory requirements
Figure 12: Evaluation of precedence scores and cosine similarity
Figure 13: Coefficient of Variation (CV) of Cis-TERN scores and sums of precedence scores across cell types
Figure 14: Perturbation analysis of peak-gene link coverage on Cis-TERN pseudotime ordering
Figure 15: Validation of Cis-TERN graph edge weights using multiome data.
Figure 16: Cancer data does not reorder healthy cells
Figure 17: Robustness of healthy cell pseudotime ordering to increasing cancer cell proportion
Figure 18: Reverse tracers make spurious visits
Figure 19: Distribution of trace lengths before termination
Figure 20: Reverse tracing of cancer cells converges rapidly

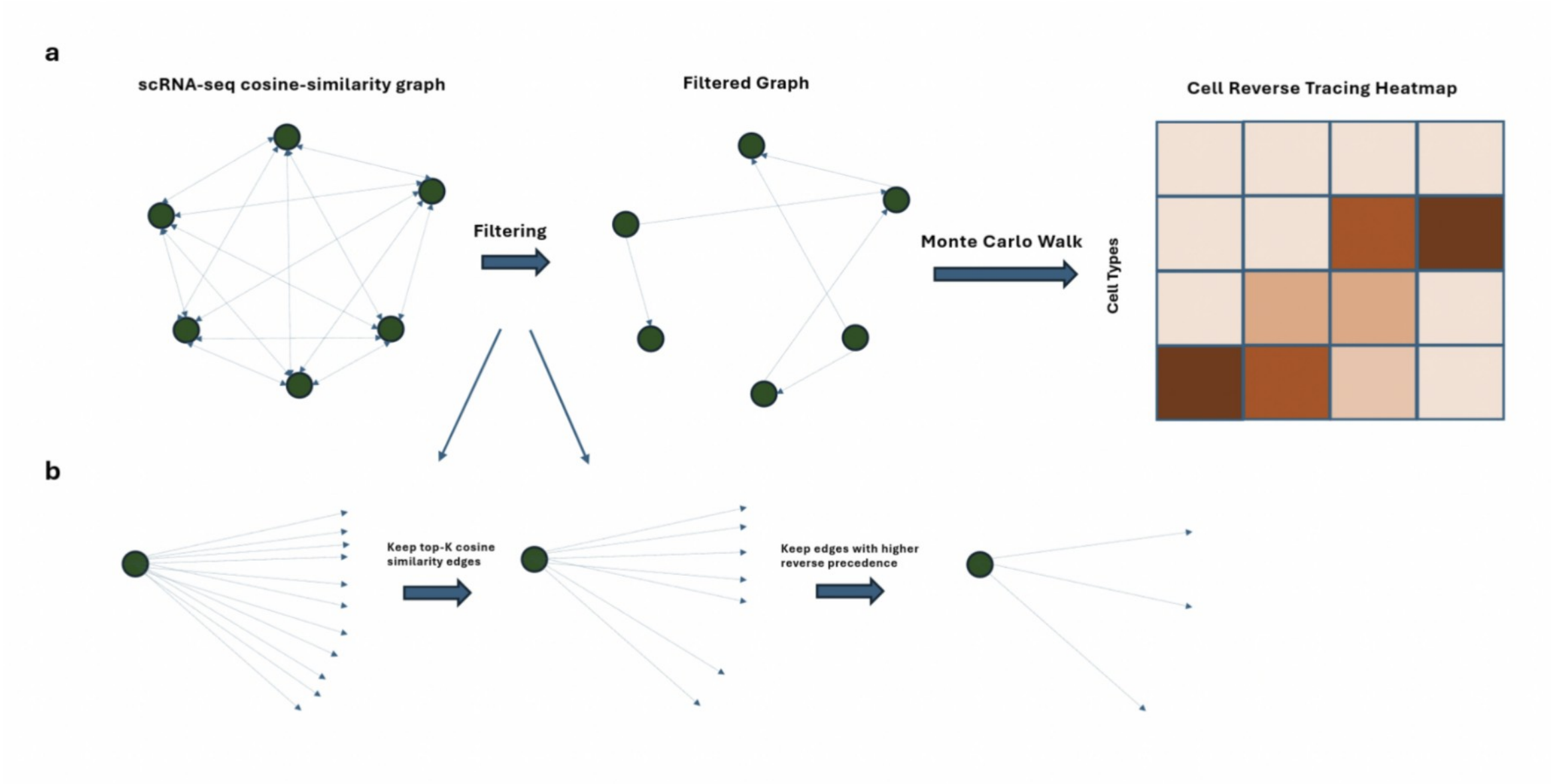

Appendix Figure 1. **Overview of reverse tracing with Cis-TERN. a)** For reverse tracing, Cis-TERN builds a pairwise cosine similarity matrix of scRNA-seq cells from which it filters edges out. It then runs a Monte Carlo simulation for up to 10 steps starting with 100 walkers on the scRNA-seq cell of interest. At each step, every walker moves according to row-normalized (row-stochastic) edge weights. **b)** Cis-TERN filters edges per cell by first keeping only the top 10% of outgoing edge weights and then removing edges to any cells that have a greater reverse Cis-TERN score.

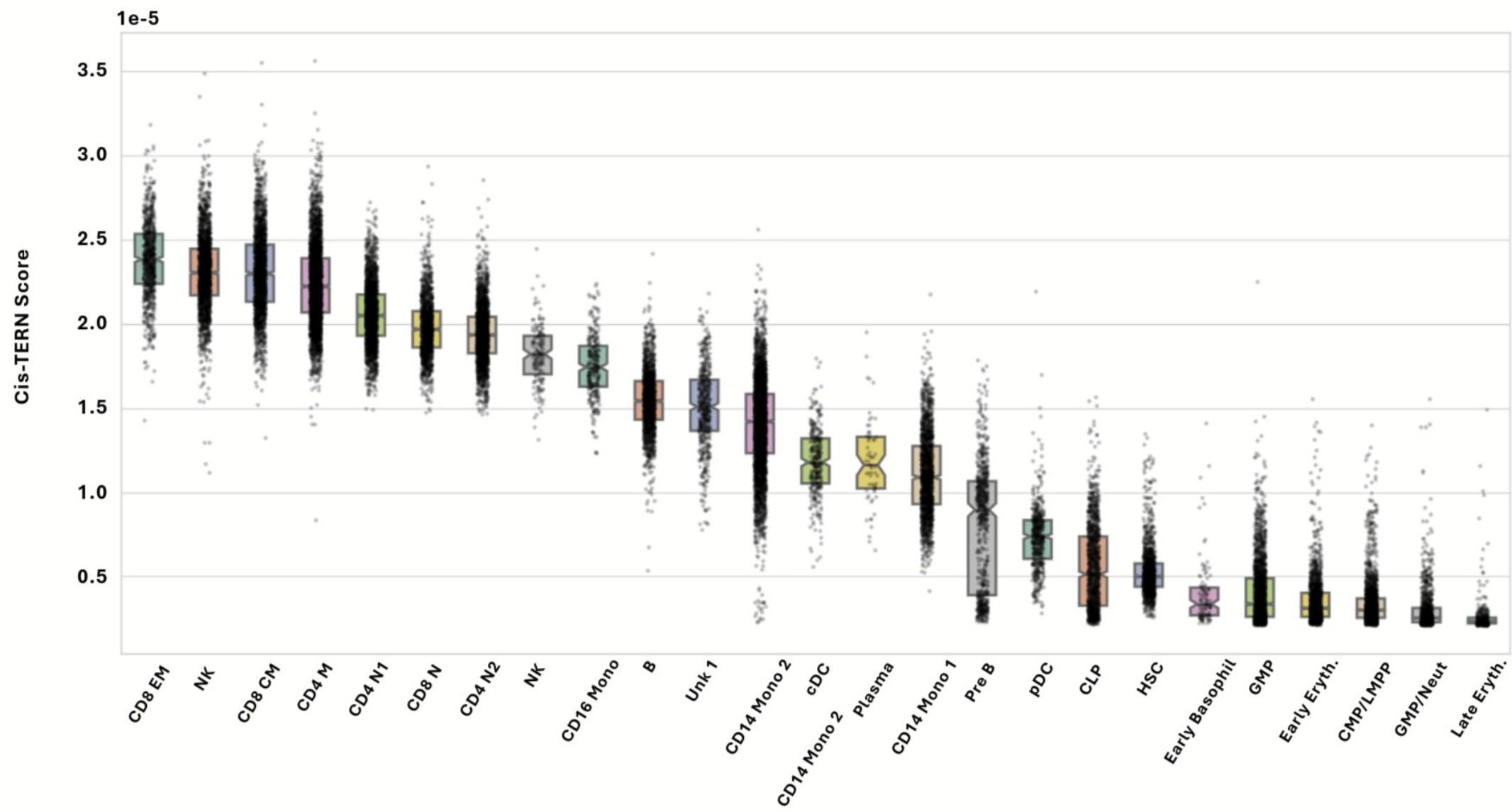

Appendix Figure 2. **Cis-TERN scores for all cell types in healthy hematopoiesis.** Notched boxplots of Cis-TERN scores for all cell types in healthy hematopoietic data ordered by median Cis-TERN score.

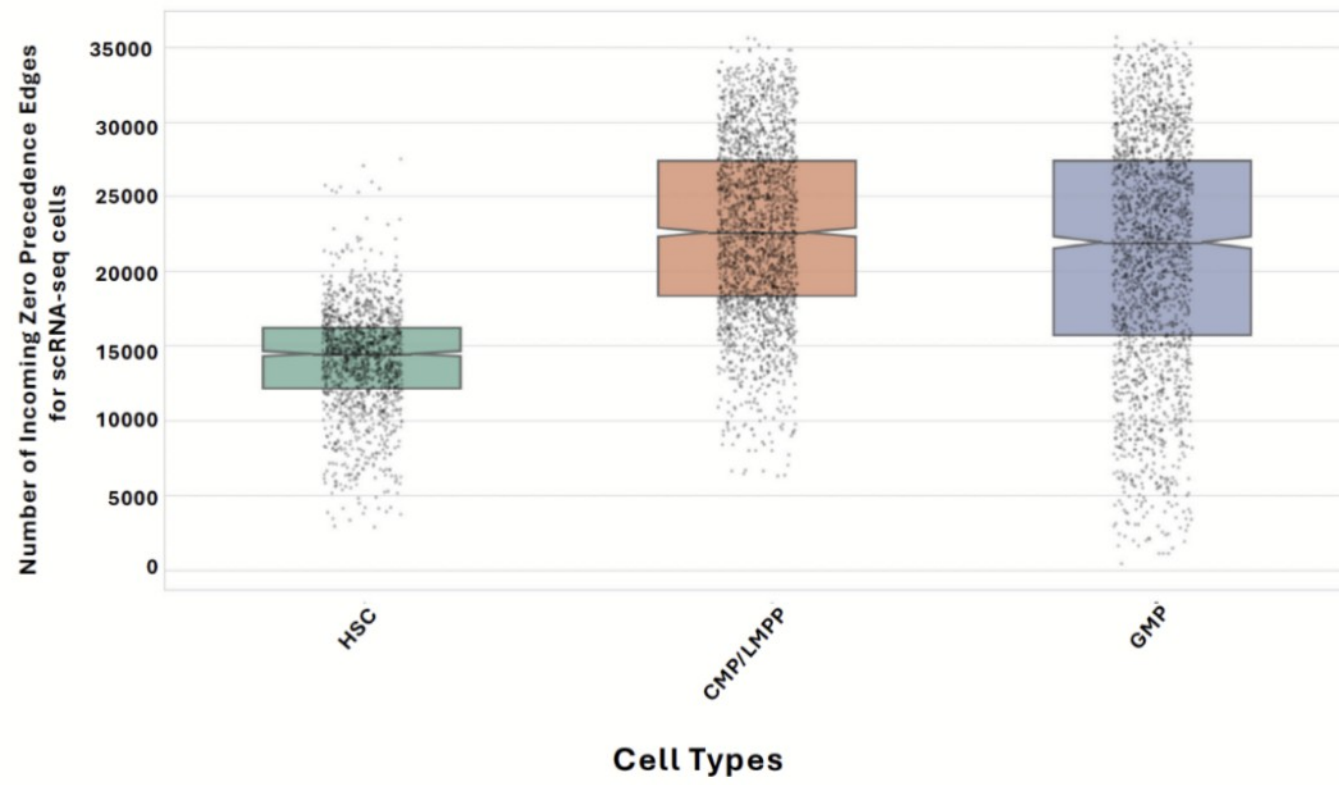

Appendix Figure 3. **HSCs have an inflated number of incoming non-zero edges.** Notched boxplots of the number of incoming zero-weighted precedence edges for scRNA-seq cells in three early progenitor cell types.

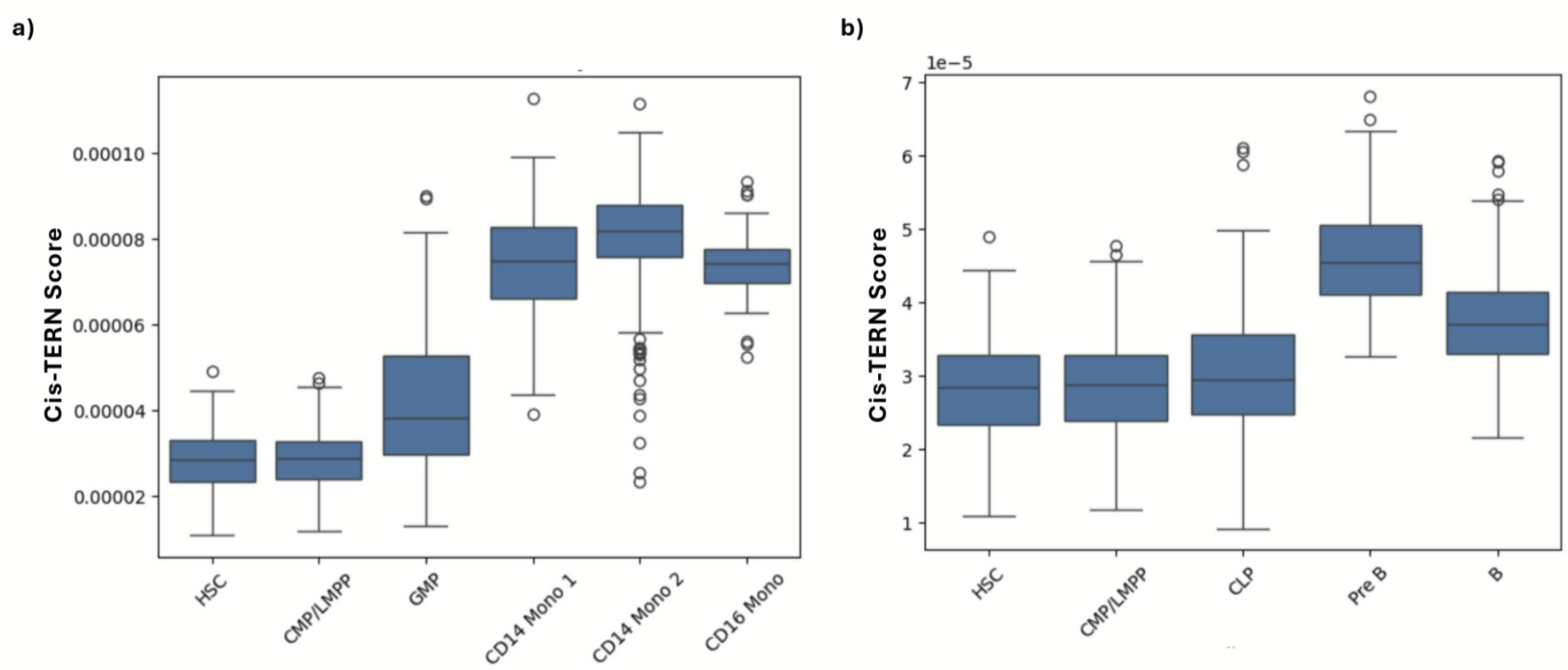

Appendix Figure 4. **Effect of peak-gene correlation threshold on Cis-TERN pseudotime ordering in hematopoiesis.** To investigate the HSC anomaly, we systematically varied the peak-gene correlation threshold used to compute pseudotime, progressively restricting the precedence score to only the strongest regulatory links. At a threshold of 0.875, HSCs are correctly placed earliest in both lineages, demonstrating that the anomaly is mechanistically linked to the inclusion of weak-peak gene links in the Cis-TERN algorithm. However, thresholds above 0.875 degrade terminal state ordering, presenting a tradeoff between correcting progenitor placement and preserving terminal state resolution. Notched boxplots of Cis-TERN pseudotime scores for cells annotated to cell types ordered by their lineages at a peak-gene correlation threshold of 0.875 applied to S_L for **a)** monocytes and **b)** B cells.

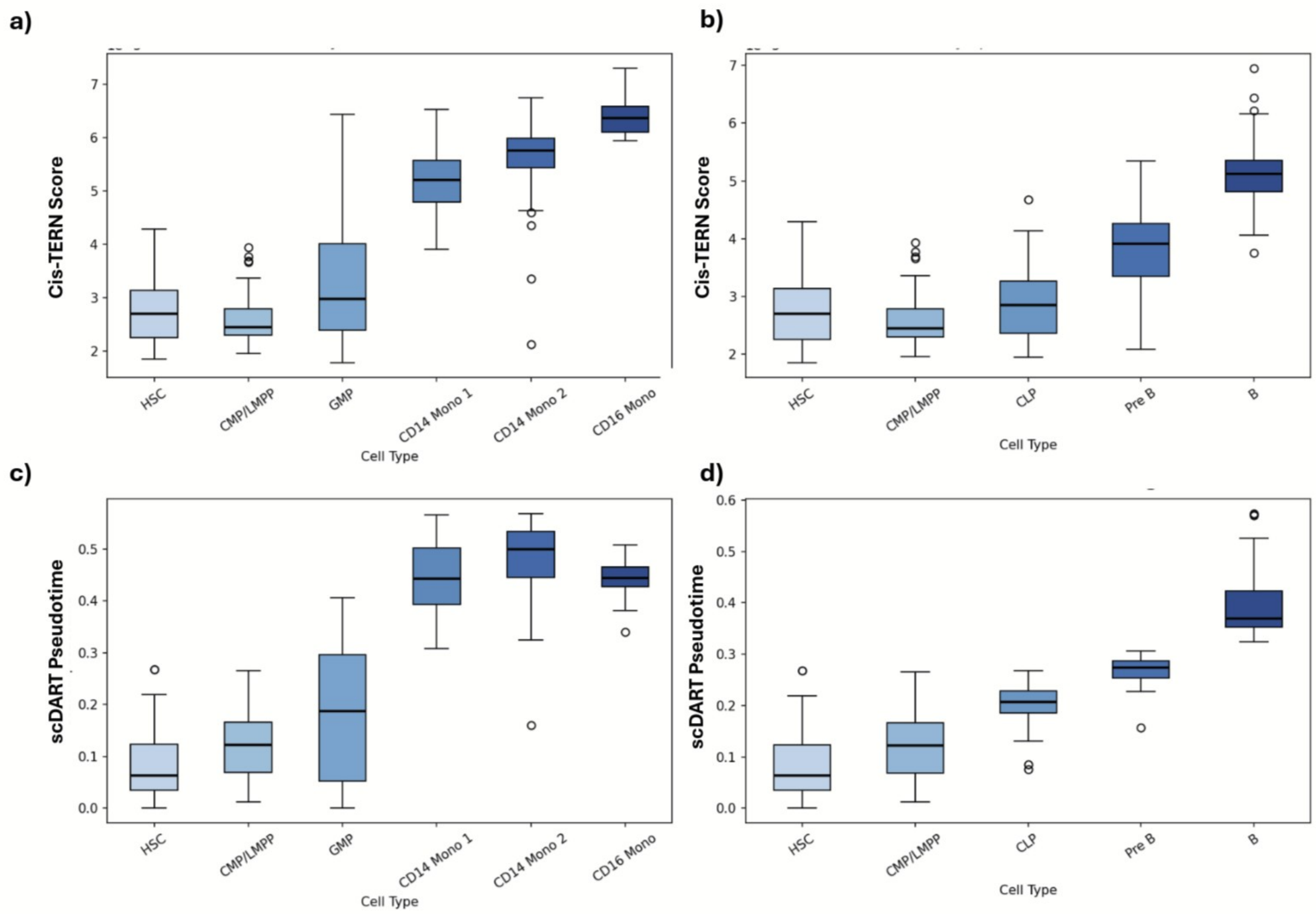

Appendix Figure 5. **Comparison of pseudotime ordering between Cis-TERN and scDART on healthy hematopoiesis data.** Notched boxplots show pseudotime scores for annotated cell types as scored by Cis-TERN in the **a)** monocyte lineage **b)** B-cell lineage and scDART for the **c)** monocyte lineage and **d)** B-cell lineage. Due to scDART's computational requirements analysis is limited to 2,000 cells per modality. HSCs are specified as the root cell for scDART.

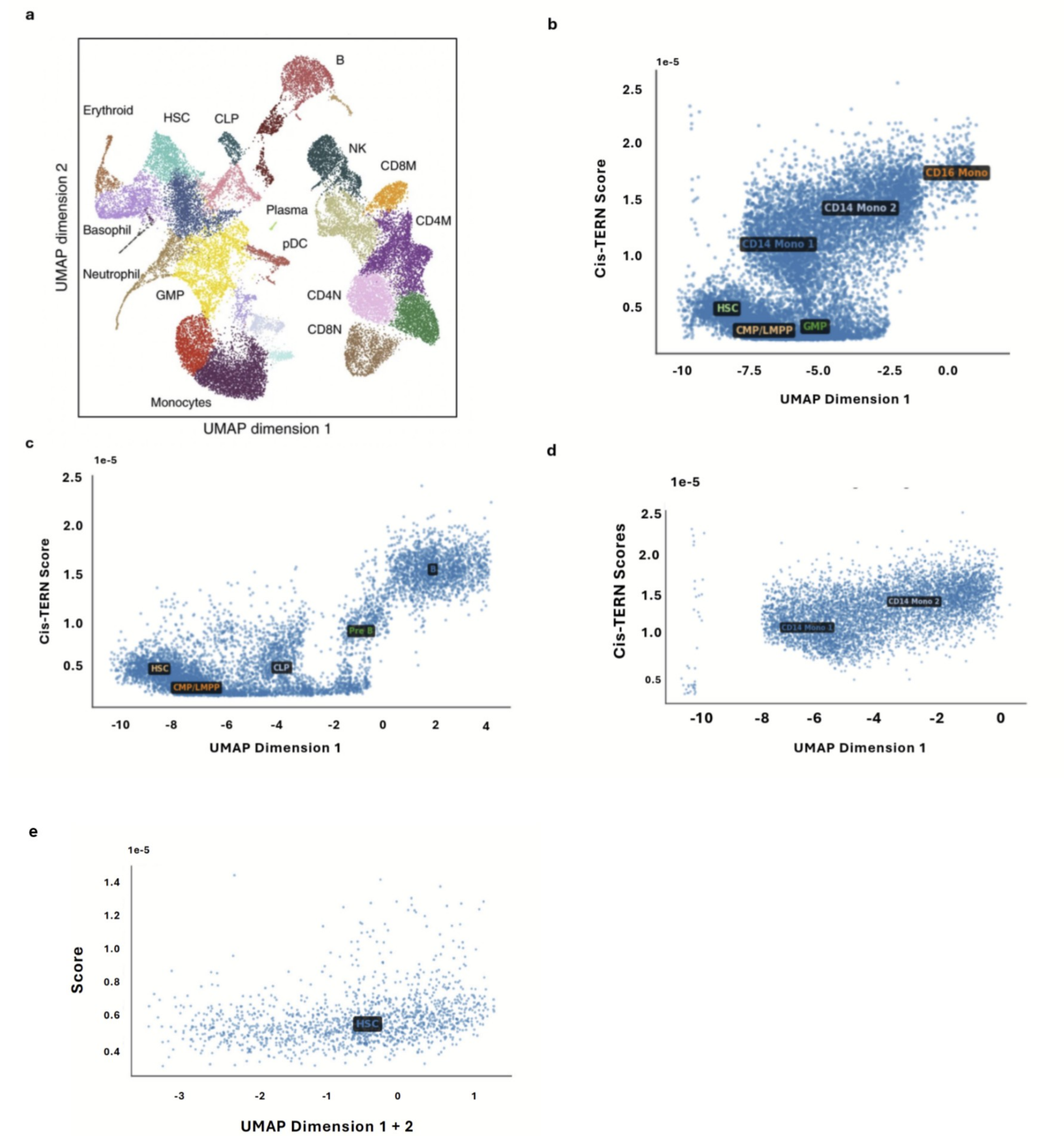

Appendix Figure 6. **Comparison of Cis-TERN scores to UMAP embedding. a)** UMAP embedding of all healthy hematopoietic scRNA-seq cells [17]. Cis-TERN score vs UMAP Dimension 1 for **b)** the monocyte cell lineage **c)** the B-cell lineage and **d)** CD14 Mono 1 and CD14 Mono 2 cells. **e)** Cis-TERN score vs UMAP Dimension 1 plus Dimension 2 for HSCs. Cell type labels appear at the centroid of all cells assigned to that cell type.

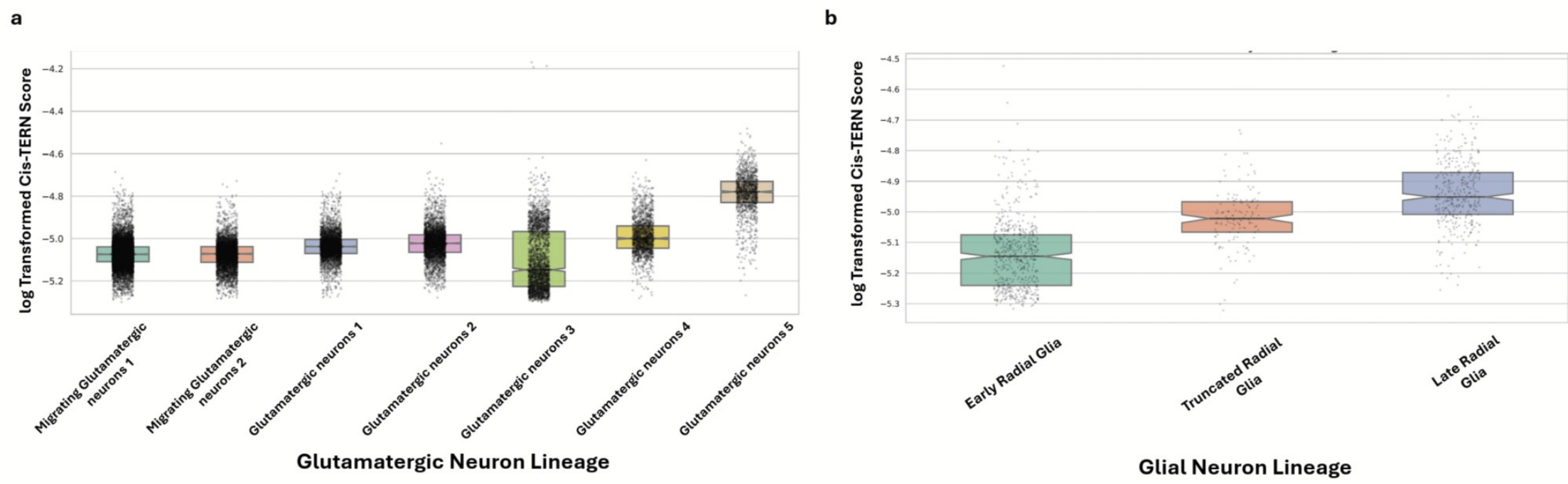

Appendix Figure 7. **Cis-TERN orders cell lineages in brain development.** Notched boxplots of Cis-TERNs scores for cells in **a)** the glutamatergic neuron lineage **b)** the radial glia lineage. For this analysis, scATAC-seq data was downloaded as fragment files from GSE162170 and scRNA-seq data was obtained from the same study's public GitHub repository as a SummarizedExperiment [21]. The SummarizedExperiment included curated cell type annotations. Overall the dataset contained 57,868 scRNA-seq cells and 31,304 scATAC-seq cells from four fetal cortex samples (PCW16, 20, 21, 24) and the scRNA-seq cells were annotated into 23 cell types. The preprocessing steps and Cis-TERN run details were the same as described in Methods section 4.4.

a)

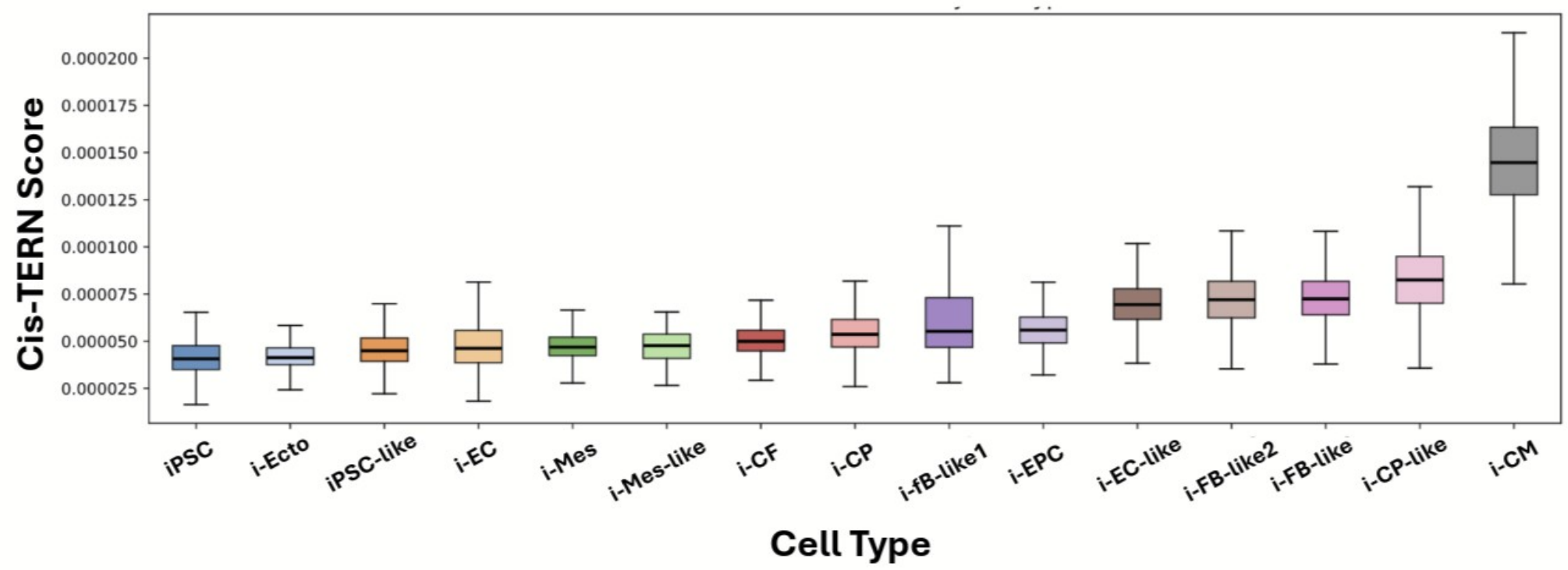

b)

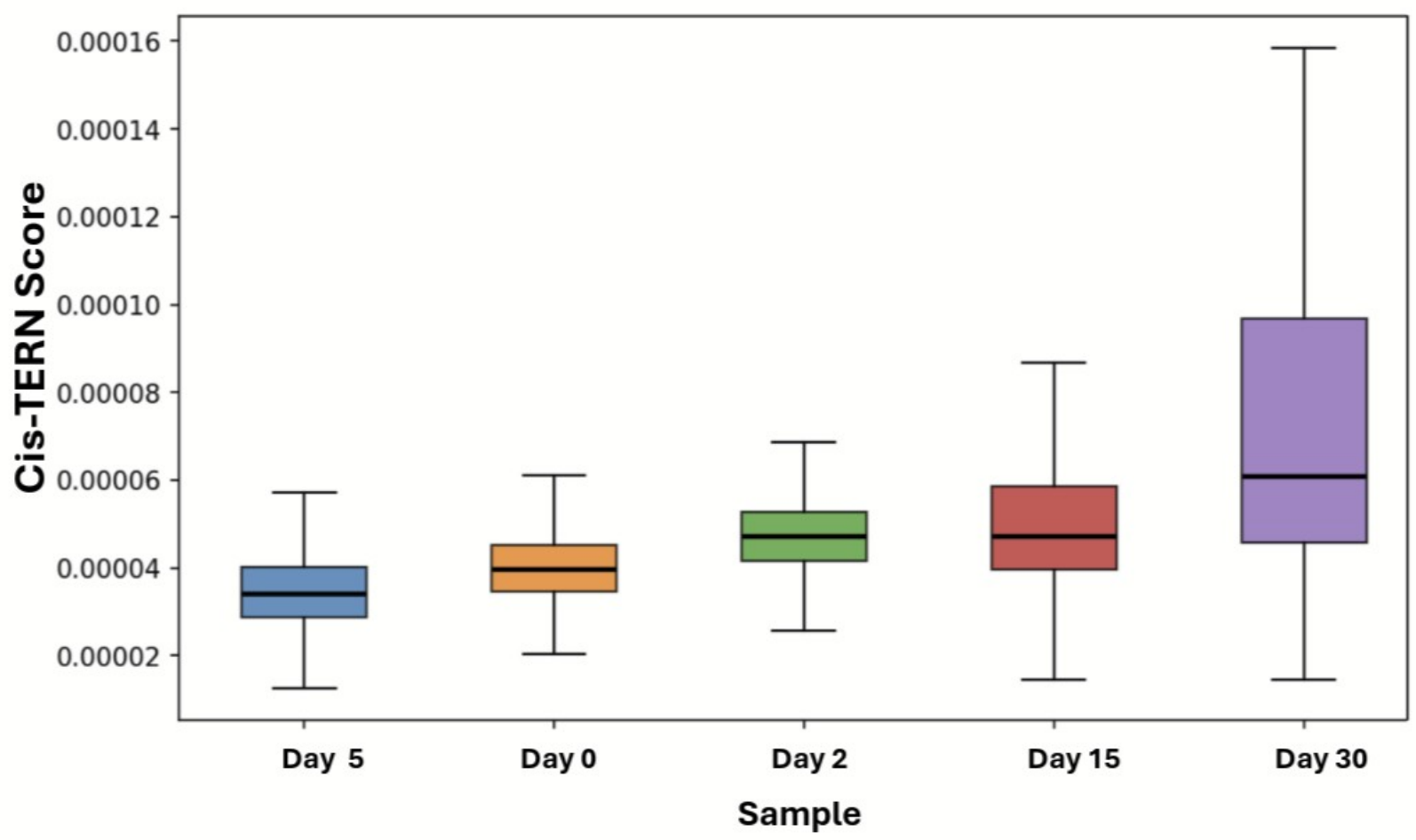

Appendix Figure 8. **Cis-TERN pseudotime scores for an in vitro cardiac differentiation time course. a)** Scores stratified by cell types ordered by known differentiation trajectory from iPSCs to mature cardiomyocytes. **b)** Scores stratified by time points. Scores broadly increase with collection day (with only the day 5 sample misordered likely due to the presence of a large number of cells labelled iPSC and iPSC-like at this time point), consistent with progressive differentiation. Peak-gene links were filtered to a stricter correlation threshold of r > 0.85 to isolate lineage-specific regulatory signals from the global accessibility patterns characteristic of iPSC chromatin states.

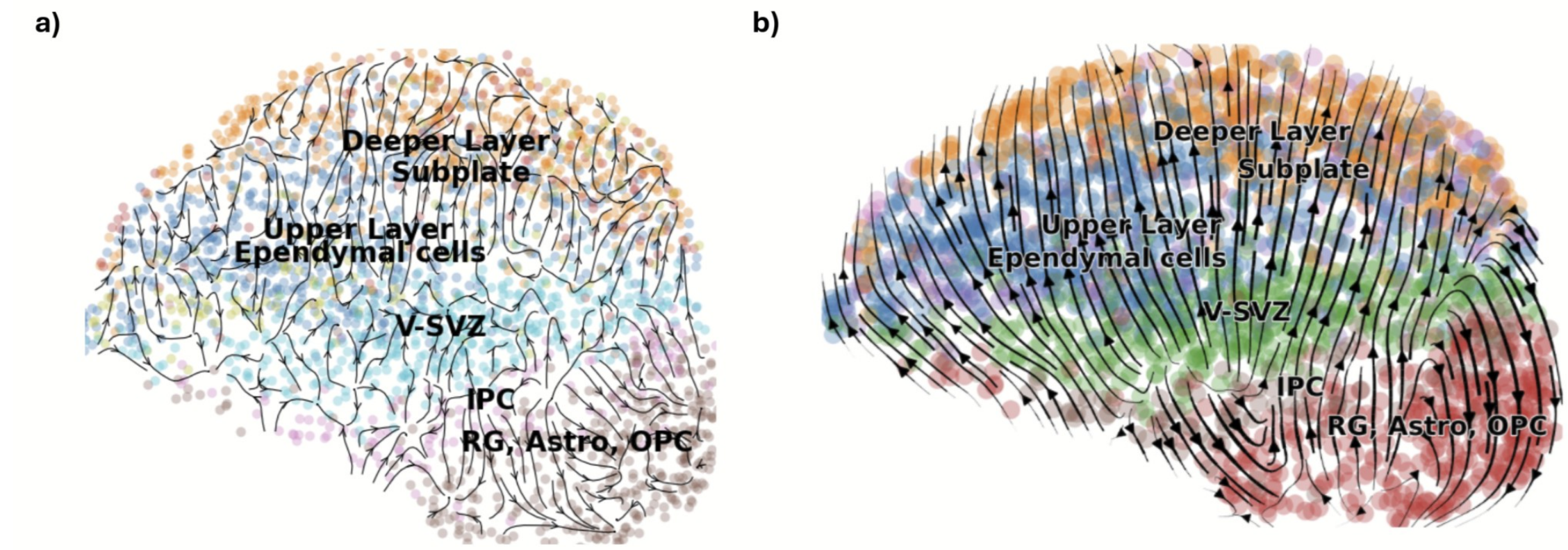

Appendix Figure 9. **Cis-TERN captures directionality in a velocity-inspired vector field. a)** Cis-TERN pseudotime scores on the developing mouse brain UMAP, with per-cell direction vectors computed by averaging the pseudotime gradient across each cell's 30 nearest neighbors in UMAP space, analogous to the neighbor-averaging approached used in standard RNA velocity visualization **b)** Visualization of MultiVelo vector field on the same dataset for comparison.

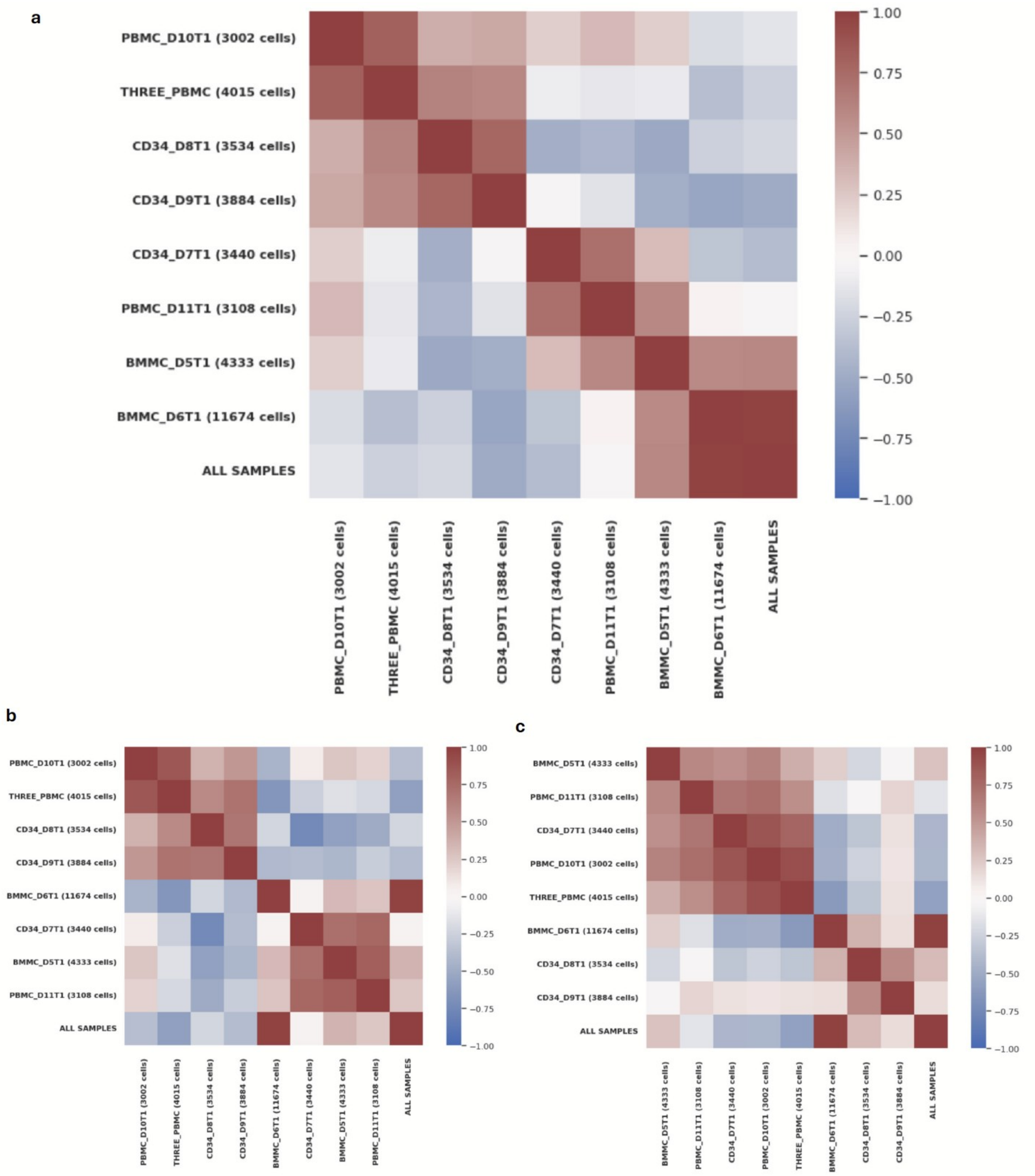

Appendix Figure 10. **Robustness of Cis-TERN to smaller quantities of scATAC-seq cells.** Heatmaps of the Pearson correlations of Cis-TERN scores for **a)** all scRNA-seq cells **b)** cells in the monocyte lineage and **c)** cells in the B-cell lineage when using only the given sample's scATAC-seq cells. While samples with more than 4,000 cells remained highly correlated, performance degraded below that point.

a)

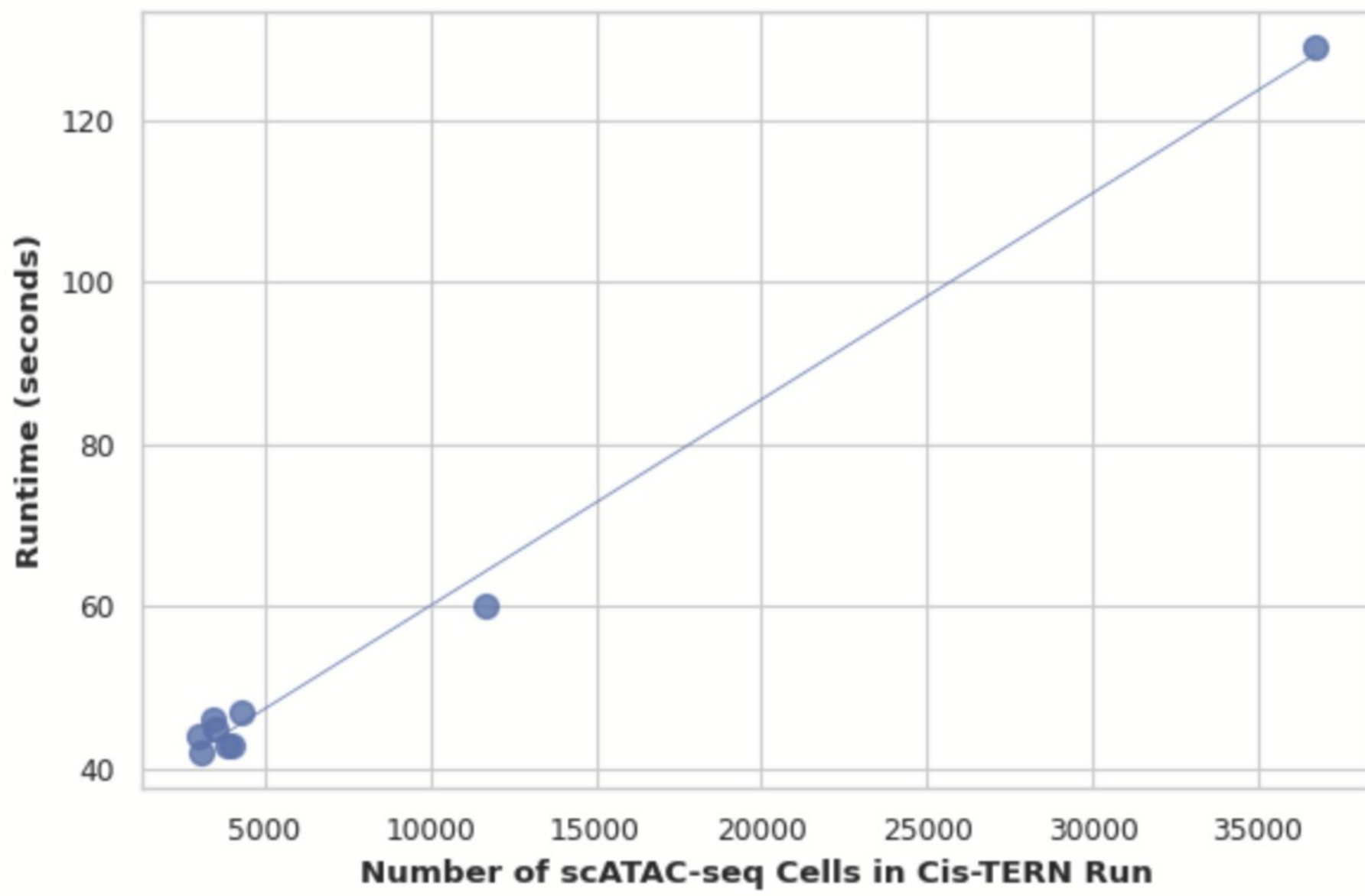

b)

Sample	Number of ATAC-seq cells	Number of RNA-seq cells	Total cells	Full Matrix (GB)	Blockwise (GB)
PBMC_D10T1	3,002	35,582	35,584	5.55	0.80
PBM_D11T1	3,108	35,582	38,690	5.58	0.82
CD34_D7T1	3,440	35,582	39,022	5.67	0.91
CD34_D8T1	3,534	35,582	39,116	5.70	0.94
CD34_D9T1	3,884	35,582	39,466	5.80	1.03
THREE_PBMC	4,015	35,582	39,597	5.84	1.06
BMMC_D5T1	4,333	35,582	39,915	5.94	1.15
BMMC_D6T1	11,674	35,582	47,256	8.32	3.09
ALL CELLS	36,990	35,582	72,572	19.62	9.81

Appendix Figure 11. **Cis-TERN runtime scaling and memory requirements. a)** Runtime of Cis-TERN in seconds for varying numbers of scATAC-seq cells. scRNA-seq cells are kept constant to 35,882. **b)** Cis-TERN's primary memory requirement comes from storing the Precedence matrix P and cosine similarity C. Rather than constructing the full dense adjacency matrix, Cis-TERN can be implemented directly on the P and C blocks, reducing memory from 19.6 GB to 9.8 GB for our full hematopoietic dataset of 72,572 cells. Memory scales linearly with the product of number of ATAC-seq and RNA-seq cells, remaining practical on standard workstation hardware for datasets of tens of thousands of cells per modality.

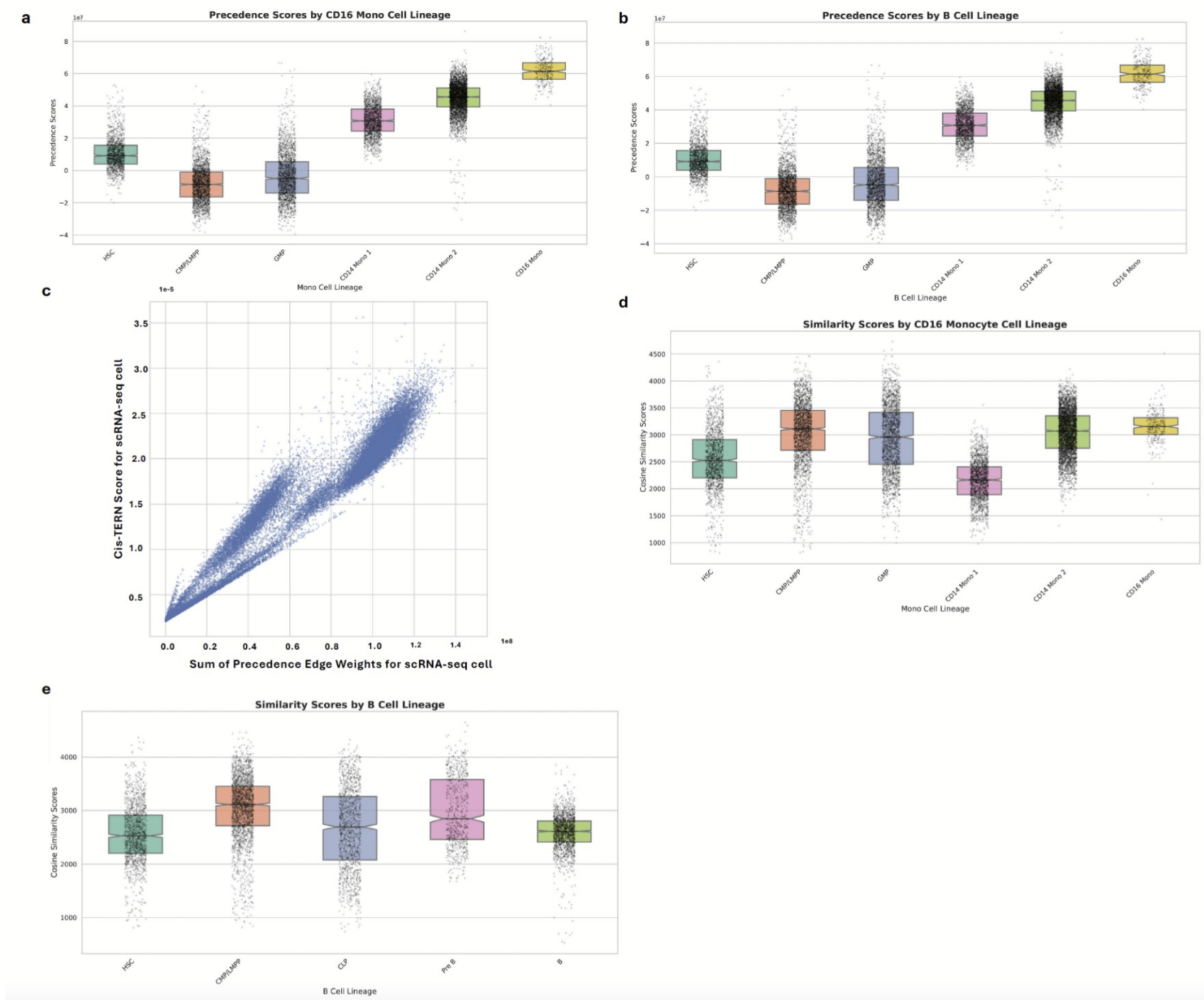

Appendix Figure 12. **Evaluation of precedence scores and cosine similarity.** Notched boxplots of the sum of incoming precedence scores for scRNA-seq cells in **a)** the monocyte lineage and **b)** the B-cell lineage. **c)** Comparison of summed precedence scores to cis-TERN scores. Notched boxplots of Cis-TERN scores when replacing precedence scores with cosine similarity in the graph for **d)** the monocyte lineage and **e)** the B-cell lineage results in a loss of ordering of cell types in each lineage.

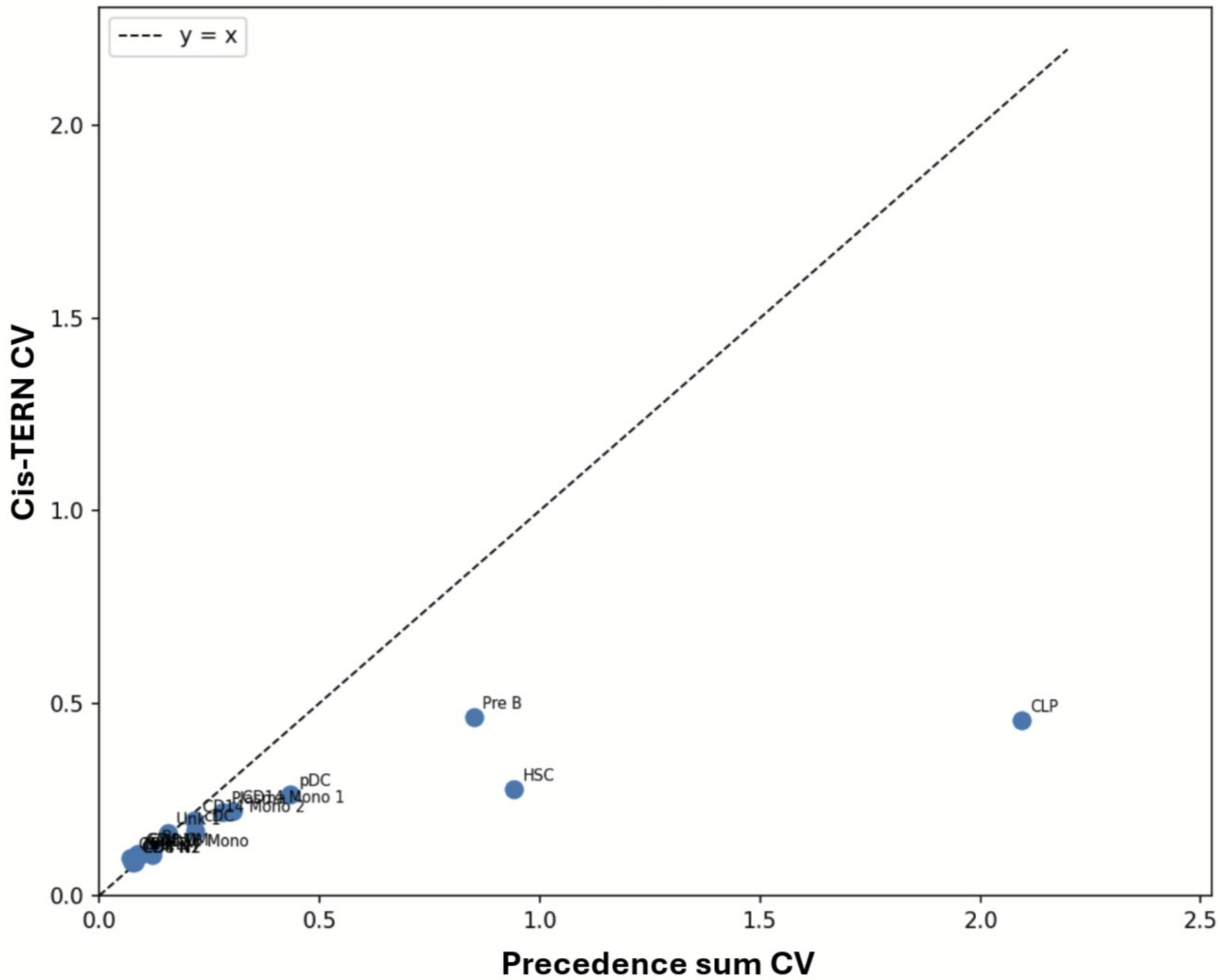

Appendix Figure 13. **Coefficient of Variation (CV) of Cis-TERN scores and sums of precedence scores across cell types.** The full model substantially reduces CV across cell types, demonstrating that cosine similarity anchoring smooths noisy precedence edges by routing random walk flow through biologically matched ATAC cells.

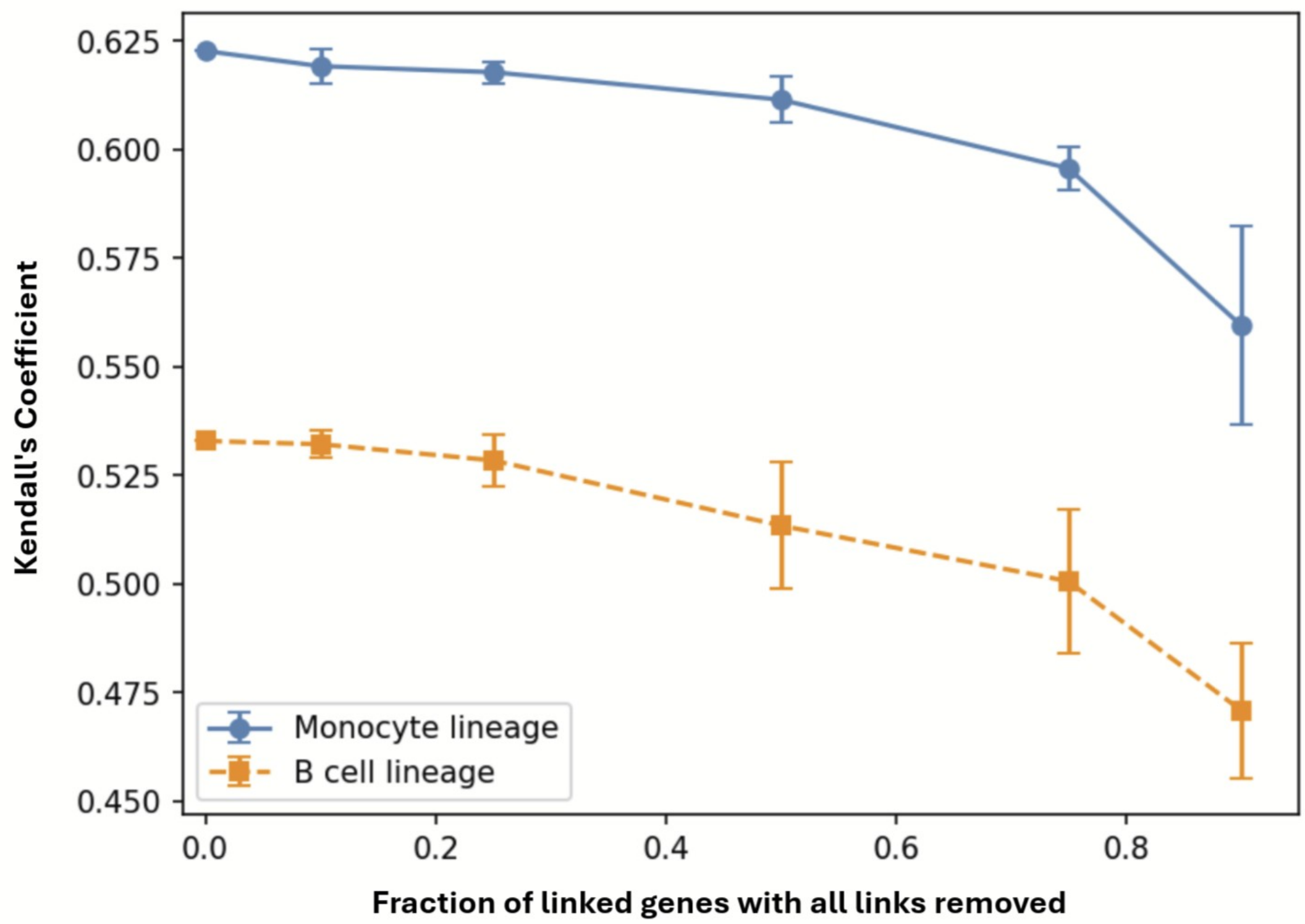

Appendix Figure 14. **Perturbation analysis of peak-gene link coverage on Cis-TERN pseudotime ordering**. To assess how gene coverage affects pseudotime accuracy, we performed a perturbation analysis where we randomly selected an increasing fraction of genes with at least one peak-gene link and removed all of their peak-gene links entirely, simulating the scenario where regulatory annotations are unavailable for a subset of genes. At each fraction, we recomputed Cis-TERN pseudotime scores and measured ordering quality using Kendall's tau against known lineage order. Both lineages show gradual degradation as gene coverage decreases.

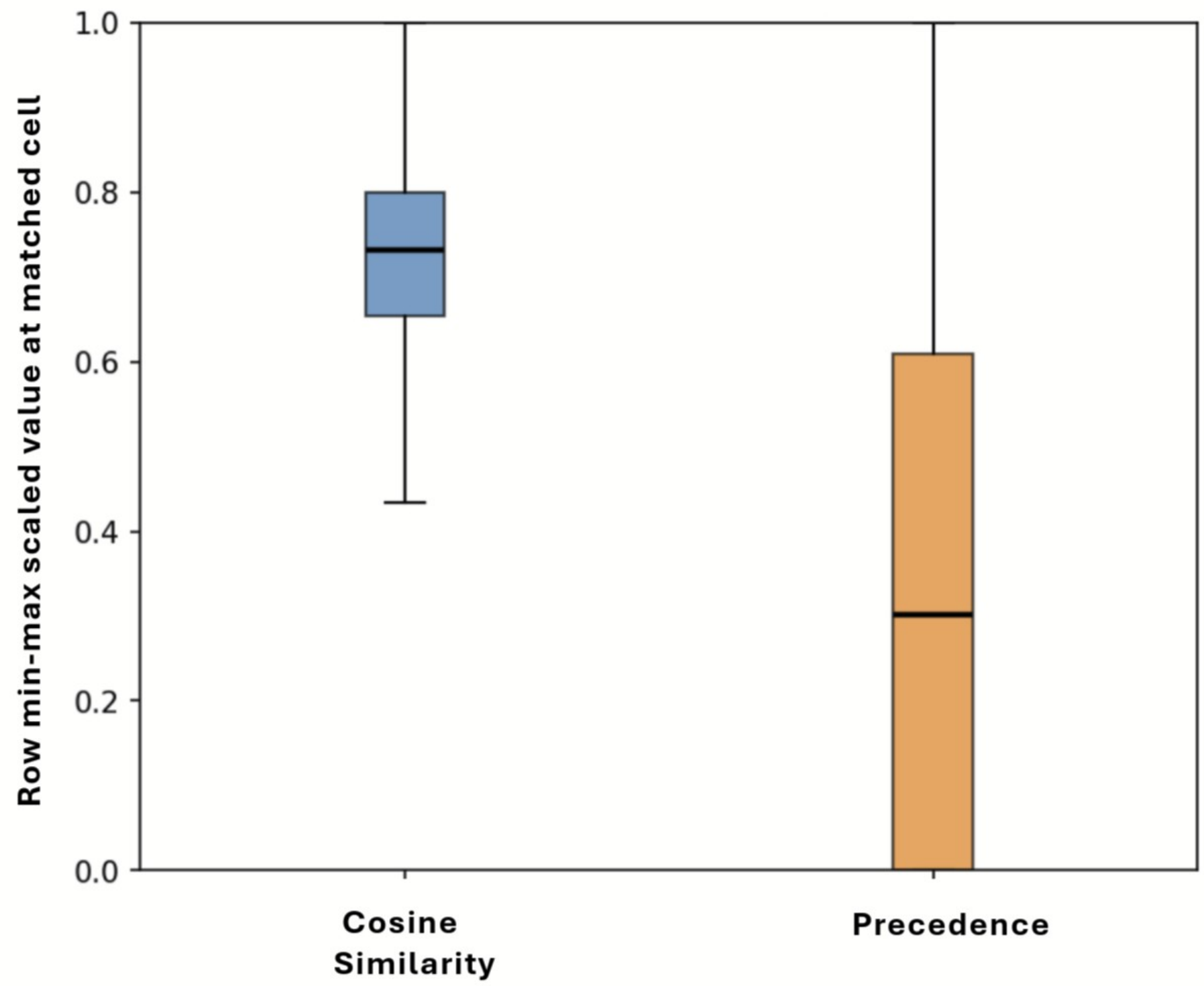

Appendix Figure 15. **Validation of Cis-TERN graph edge weights using multiome data.** In the multiome setting, each RNA cell has a known corresponding ATAC cell, providing a ground-truth cross-modal pairing. Using Mouse Brain E18 multiome data, for each matched RNA-ATAC cell pair, we extracted the cosine similarity score (encoding cross-modal similarity) and the precedence score (encoding temporal offset between distal chromatin accessibility and gene expression) from their respective matrices, applying row-wise min max normalization see how close the matching cell in the other modality was to the maximum score. As expected, we observe that the matching cell is typically among the highest cosine similarity edges but not the precedence score edges. This is the expected result as multiome measurements are taken simultaneously from the same cell, the RNA and ATAC gene profiles should be highly similar (high cosine similarity), but other cells may have a closer temporal relationship encoded in distal accessibility. A high precedence score indicates that one cell's chromatin state precedes another cell's expression, a signal that is expected between cells at different developmental stages, not within a single cell at one moment in time.

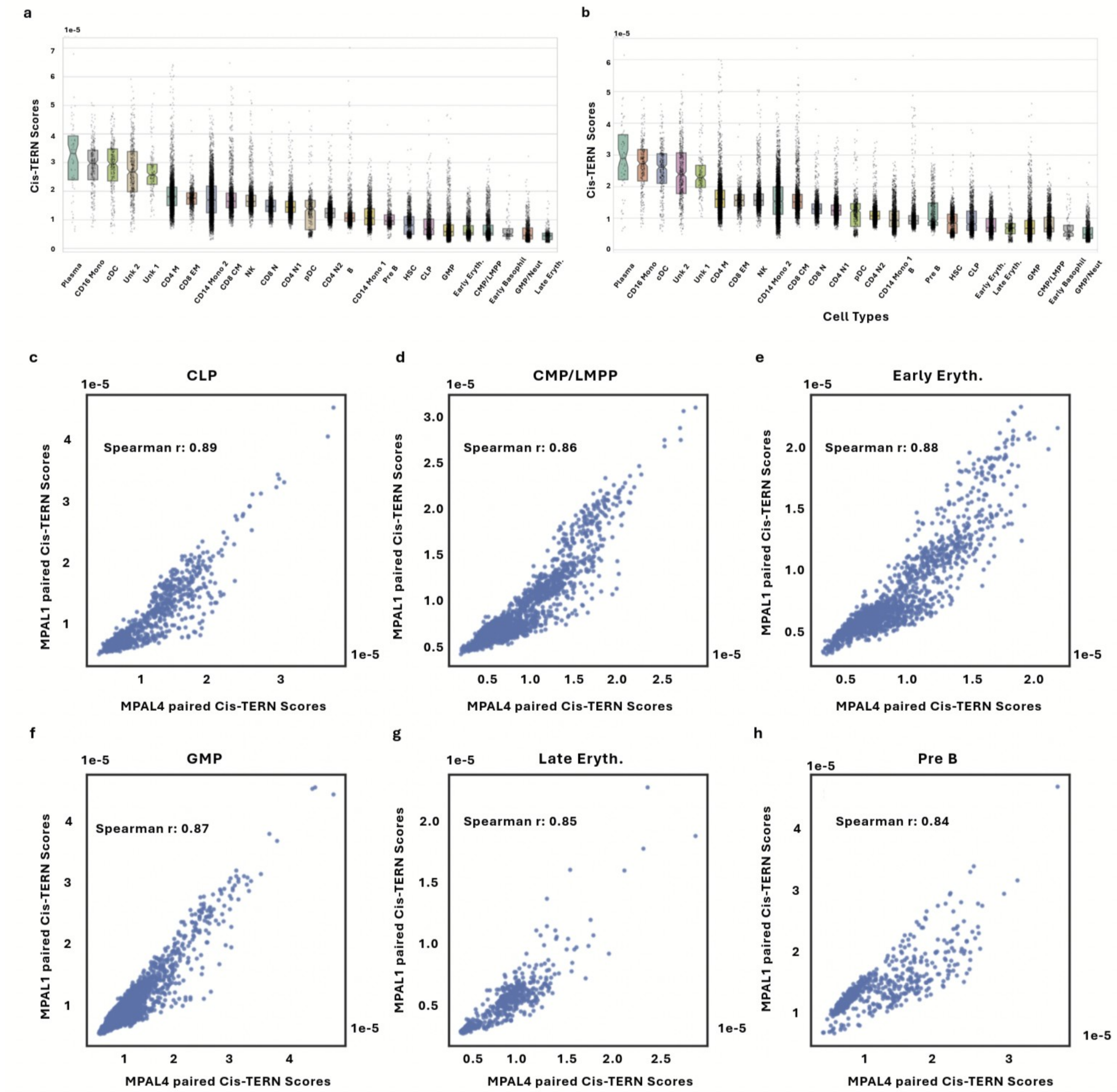

Appendix Figure 16. **Cancer data does not reorder healthy cells.** Cis-TERN scores for healthy cell types when paired with **a)** MPAL1 (T-myeloid MPAL) **b)** MPAL4 (B-myeloid MPAL). Per cell type, cis-TERN scores were highly correlated between MPAL1 and MPAL4 with all Spearman's ρ > 0.9 except for **c)** CLP cells, **d)** CMP/LMPP cells, **e)** early erythrocytes, **f)** GMP cells, **g)** late erythrocytes, and **h)** pre-B cells, all of which had Spearman's ρ > 0.8.

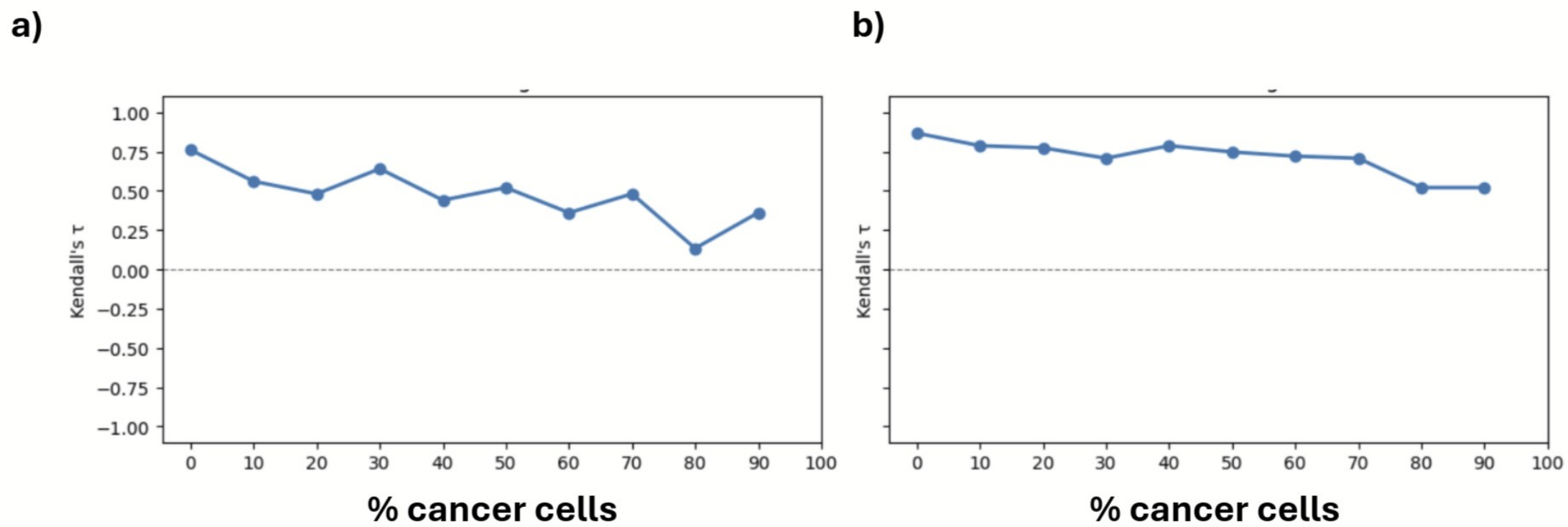

Appendix Figure 17. **Robustness of healthy cell pseudotime ordering to increase cancer cell proportion.** Kendall's tau between Cis-TERN pseudotime scores and known lineage order is shown for the **a)** B-cell and **b)** Monocyte lineages as the proportion of MPAL cancer cells in the graph is varied from 0% to 90% in 10% increments across both scRNA-seq and scATAC-seq modalities simultaneously.

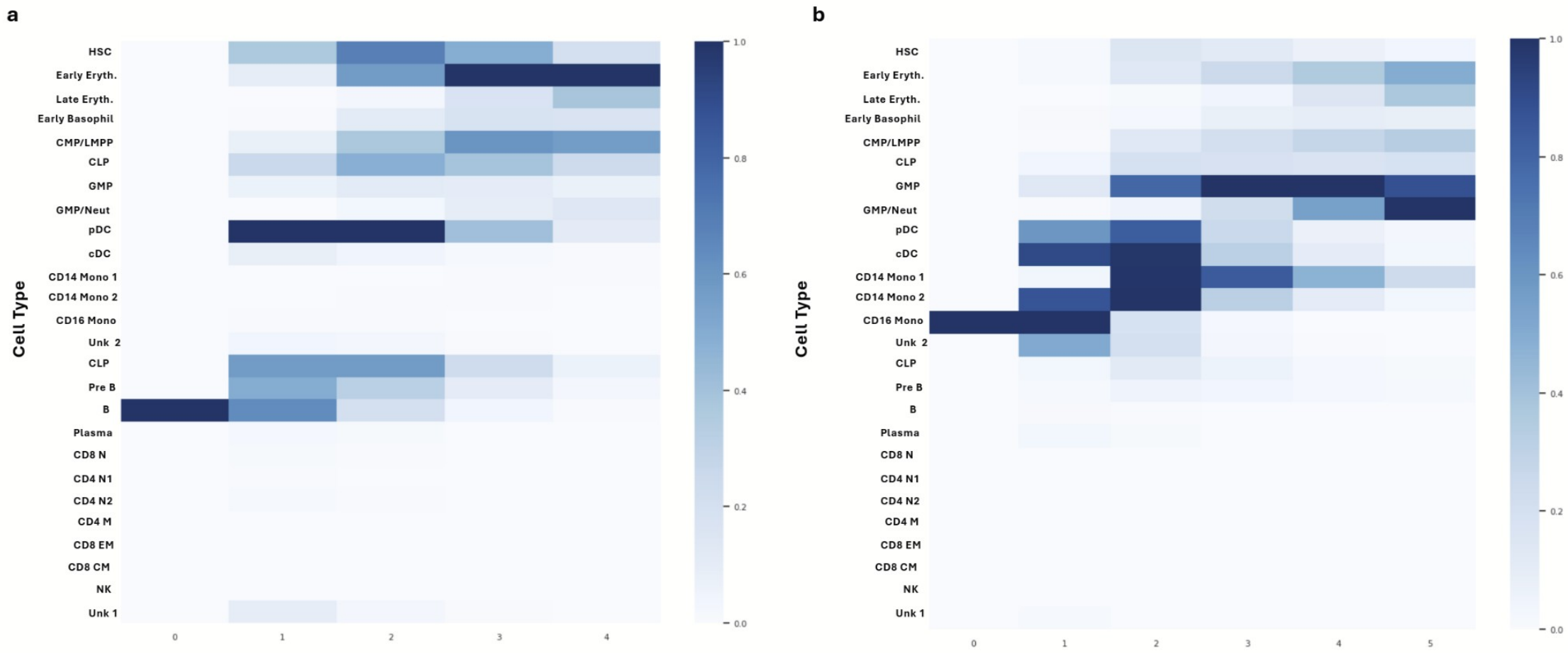

Appendix Figure 18. **Reverse traces make spurious visits.** The min-max scaled fraction of traces that visited each cell type when traces started at **a)** B cells and **b)** CD16 monocytes. The traces make additional spurious visits to cell types outside of the known lineages.

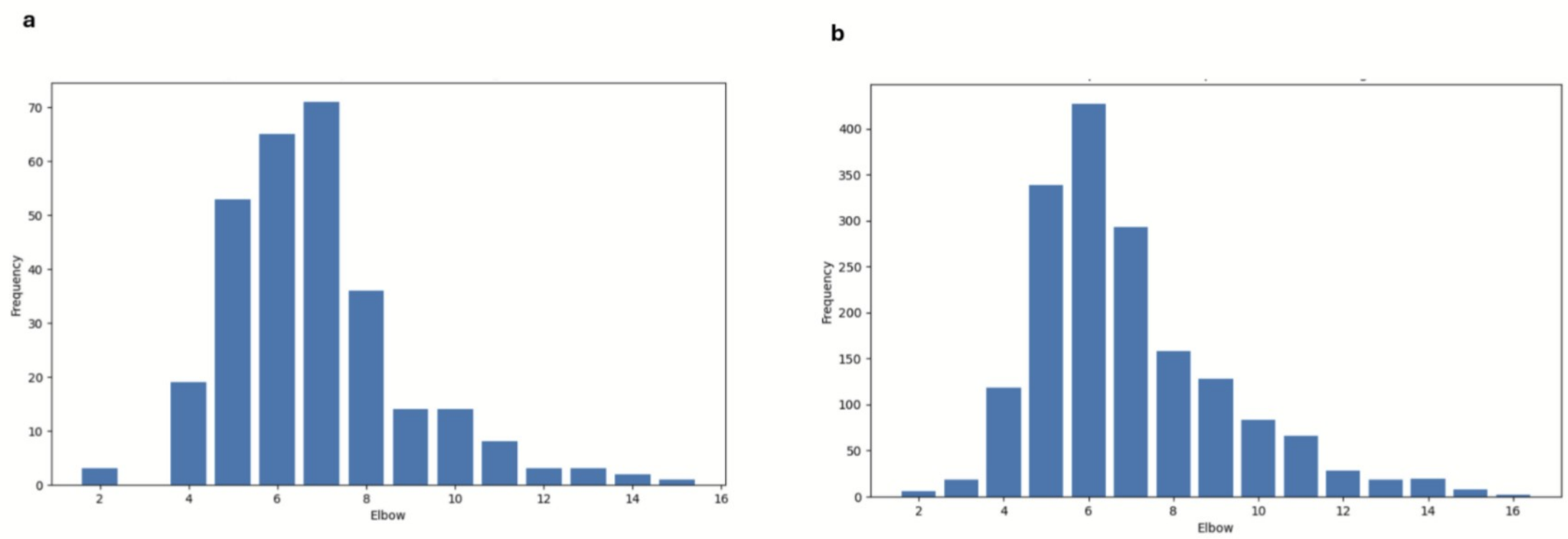

Appendix Figure 19. **Distribution of trace lengths before termination.** The distribution of optimal walk steps for reverse tracing of **a)** all CD16 Mono cells and **b)** all B cells based on elbow method termination.

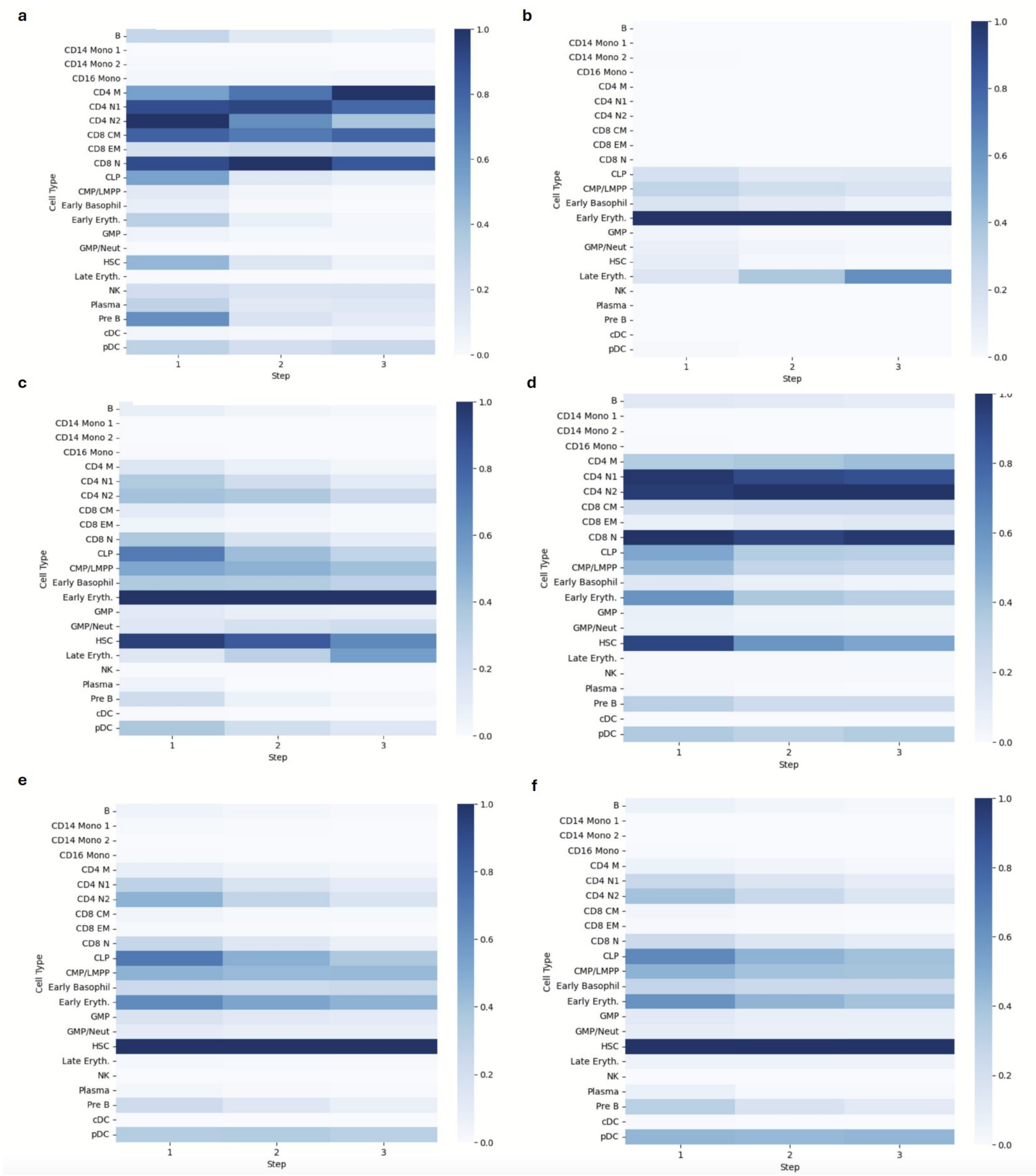

Appendix Figure 20: **Reverse tracing of cancer cells converges rapidly.** Cis-TERN reverse tracing results for starting with all cancer cells at step 0 for **a)** MPAL1, **b)** MPAL2, **c)** MPAL3, **d)** MPAL4, **e)** MPAL5, and **f)** MPAL5R converge after three steps.
